# A Cost-Effective Semi-Ab Initio Approach to Model Relaxation in Rare-Earth Single-Molecule Magnets

**DOI:** 10.1021/acs.jpclett.1c02367

**Published:** 2021-09-07

**Authors:** Elena Garlatti, Alessandro Chiesa, Pietro Bonfà, Emilio Macaluso, Ifeanyi J. Onuorah, Vijay S. Parmar, You-Song Ding, Yan-Zhen Zheng, Marcus J. Giansiracusa, Daniel Reta, Eva Pavarini, Tatiana Guidi, David P. Mills, Nicholas F. Chilton, Richard E. P. Winpenny, Paolo Santini, Stefano Carretta

**Affiliations:** † Universitá di Parma, Dipartimento di Scienze Matematiche, Fisiche e Informatiche, 43124 Parma, Italy; ‡ UdR Parma, INSTM, I-43124 Parma, Italy; ¶ Department of Chemistry, The University of Manchester, Oxford Road, Manchester M13 9PL, U.K.; § Frontier Institute of Science and Technology, Xi’an Jiaotong University, 99 Yanxiang Road, 710054 Xi’an, Shaanxi, China; ∥ Institute for Advanced Simulations, Forschungszentrum Juelich, 52428 Juelich, Germany; ∞ JARA High-Performance Computing, RWTH Aachen University, 52062 Aachen, Germany; ⊥ ISIS Facility, Rutherford Appleton Laboratory, Didcot OX11 0QX, U.K.

## Abstract

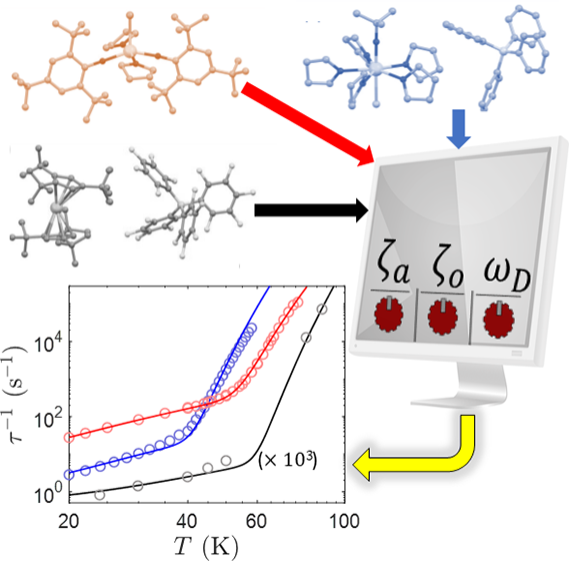

We discuss a cost-effective approach to understand magnetic relaxation in the new generation of rare-earth single-molecule magnets. It combines ab initio calculations of the crystal field parameters, of the magneto-elastic coupling with local modes, and of the phonon density of states with fitting of only three microscopic parameters. Although much less demanding than a fully ab initio approach, the method gives important physical insights into the origin of the observed relaxation. By applying it to high-anisotropy compounds with very different relaxation, we demonstrate the power of the approach and pinpoint ingredients for improving the performance of single-molecule magnets.

The intense research on the new generation of 4f-based single-molecule magnets (SMMs)^1,2^ with strong axial anisotropy^3−16^ has recently culminated in the identification of dysprosocenium complexes,^17−19^ which display blocking temperatures that surpass liquid nitrogen temperatures. This sudden jump could open the possibility to reach even higher working temperature if the main ingredients behind such performances are well understood. It is evident that the anisotropy barrier *U*_eff_ is not the only figure of merit,^20^ since 4f-based molecules with similarly high barriers can display very different relaxation dynamics.^21−24^ Hence, a sound and efficient method explaining the very different phonon-induced relaxation in molecular crystals of different high-barrier molecules is needed to make a step forward.^20,25,26^ A fully ab initio approach^27,28^ would yield the maximum insight into the origin of magnetic relaxation of specific compounds, but the huge amount of computational resources needed would strongly limit the applicability of such a method to only a small number of compounds.

To design a cost-effective theoretical approach, we need to balance the amount of predictive power with the resources required for numerical calculations. Here we describe such an approach and show that it explains why molecular crystals of different 4f SMMs with similar anisotropy barriers are characterized by very different phonon-induced relaxation. Remarkably, the approach proposed yields physical insights that cannot be obtained with the phenomenological models widely used for fitting relaxation rates, whose parameters are not connected to microscopic Hamiltonians. At the same time, it is not as demanding as fully ab initio calculations since it takes advantage of these techniques only to calculate the key ingredients of the Hamiltonian. As a preliminary step, we recently applied this approach to a protypical dysprosocenium system ([Dy(C_5_H_2_^*t*^Bu_3_-1,2,4)_2_][B(C_6_F_5_)_4_] (**1**, Figure 1a)).^29^ Here we finally demonstrate the reliability and power of this method by successfully applying it to two benchmark Dy-based compounds, both of which are characterized by high anisotropy barriers but have very different and much faster relaxation than **1**: the pentagonal bipyramidal [Dy(^*t*^BuO)Cl(THF)_5_][BPh_4_]·2THF complex (**2**, Figure 1b)^30^ and the five-coordinate [Dy(Mes*O)_2_(THF)_2_Br] complex (Mes* = 2,4,6-tri-*tert*-butylphenyl) (**3**, Figure 1c).^24^ Crystalline batches of **2**(30) and **3**(24) were synthesized according to literature procedures to make pure samples; single-crystal XRD was used to check the unit cells of multiple crystals to confirm that the identities of these samples matched literature data (see CCDC 1450752 and 1978054). Complementary characterization data were provided by the preparation of the structurally analogous diamagnetic Y(III) analogues of **2** (**2**-Y)^30^ and **3** (**3**-Y), which were additionally studied by ^1^H and ^13^C NMR spectroscopy to assess the bulk purity of samples. Yttrium complexes are often used to provide diamagnetic matrices for doping studies of late lanthanide SMMs, where increased distances between paramagnetic ions reduce dipolar interactions and help distinguish magnetic relaxation mechanisms. Full synthetic and crystallographic details are compiled in the Supporting Information.

**Figure 1:**
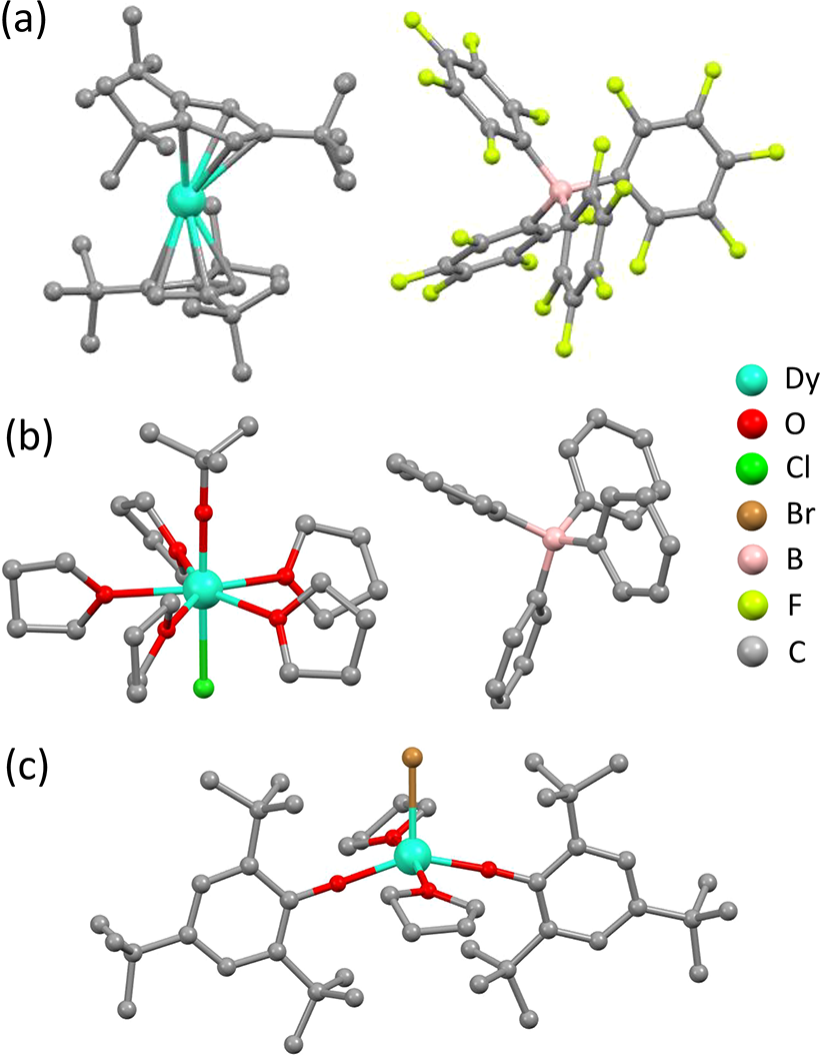
Molecular structures of (a) **1**, (b) **2**, and (c) **3**; hydrogen atoms have been omitted for clarity.

An important ingredient of our approach is the phonon density of states (pDOS), ρ(*E*), which we obtain ab initio. To put this calculation to the test, we probed it with Inelastic Neutron Scattering (INS), finding excellent agreement. Then, via our approach we calculate the temperature-dependence of the relaxation rates, reproducing the experimental data with a very good agreement for all the compounds. Furthermore, we highlight the main ingredients that make the Raman mechanism much more efficient in **2** and **3** and thus considerably worsen their performance as SMMs with respect to that of **1**.

The Hamiltonian describing molecular crystals of these SMMs is
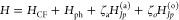
1where the first term contains the crystal field (CF) splitting ∑_*k*,*q*_*B*_*k*_^*q*^*O*_*k*_^*q*^ of the *J* = 15/2 angular momentum multiplet for each Dy^3+^ ion in the crystal, with *O*_*k*_^*q*^ being Steven’s operators equivalents.^31^ The *H*_ph_ term in eq 1) is the phononic Hamiltonian, and *H*_*Jp*_^(*a*,*o*)^ describes magnetoelastic coupling with acoustic and optical phonons (parametrized by ζ_*a*_ and ζ_*o*_). The CF parameters can be reliably calculated ab initio using complete active space self-consistent field spin–orbit (CASSCF-SO) calculations with MOLCAS 8.0,^24,30,32^ and *H*_ph_ can be obtained via DFT (PBE) calculations using Quantum Espresso.^33^ The most demanding parts of ab initio calculations are magnetoelastic interactions. However, this huge task is not really necessary. On the one hand, Raman processes are dominated by dispersive “acoustic” modes, and *H*_*Jp*_^(*a*)^ can be reasonably approximated by applying a “rotational Debye model”^34^ and using the DFT (PBE) pDOS.^29,35−37^ Indeed, the energy integral relevant for Raman processes washes out details of individual phonon modes (see below, eq 2). On the other hand, only high-energy nondispersive optical phonons are involved in the high-temperature Orbach regime^38,39^ and *H*_*Jp*_^(*o*)^ can thus be approximated by simpler calculations in the gas phase (see the Supporting Information for more details). It is important to stress that in molecular crystals of SMMs such as **1**, **2**, and **3**, many low-energy optical modes typically correspond to collective vibrations with little bond stretching and are significantly dispersive (see the Supporting Information for more details). Thus, the effective upper energy limit for the dispersive modes described via the rotational Debye model, ℏω_*D*_, is here treated as a fitting parameter.

Indeed, in our approach only three free Hamiltonian parameters (ζ_*a*_, ζ_*o*_, and ω_*D*_) are needed to model phonon-induced relaxation and are determined by a comparison with relaxation measurements. All the other quantities in eq 1 are efficiently calculated ab initio, with the pDOS and lowest CF energies also being independently validated by the comparison with targeted inelastic neutron scattering experiments. At last, the low-temperature relaxation of 4f-based SMMs is generally dominated by temperature-independent quantum tunneling processes, which can therefore be modeled by a constant relaxation rate. Therefore, we will focus on phonon-driven relaxation in the intermediate and high temperatures regimes.

The intermediate temperature range is crucial in determining blocking temperatures.^29^ In this regime, the system dynamics is restricted to the lowest Kramers doublet, and excited CF states only contribute via nonresonant Raman processes. The corresponding relaxation rate is proportional to^29,35,40^
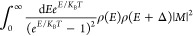
2where ρ(*E*) is the pDOS computed by DFT, Δ is the (practically negligible) Zeeman gap between the two states of the ground doublet, and *M* contains matrix elements of the magnetoelastic term *H*_*Jp*_^(*a*,*o*)^. Although the integral in eq 2 is calculated over the whole phonon spectrum, due to the form of its integrand, the Raman relaxation is driven mainly by low-energy dispersive modes (in the present model, those with energy *E* ≤ ℏω_*D*_).^29^ By numerical trapezoidal integration, we obtain from eq 2 the Raman contribution to the relaxation rate. This, in general, does not follow a power-law *T*-dependence apart from a rather narrow intermediate temperature range (see Figure 3b and comments below). Nonetheless, extracting “effective” power-law behaviors in specific temperature regimes can be useful for a quick comparison with the experimental literature, where often such a power-law fit is adopted to interpret the data. Hence, in the following we calculate the Raman contribution to the relaxation rate from eq 2 and use it to compare the experimental data in the whole *T* range. In addition, we fit the calculation with an effective power-law *T*-dependence τ_Raman_^–1^ = *CT*^*n*^ in a properly narrow temperature range. This allows us to directly extract the two parameters governing the relaxation rate in the Raman regime: the prefactor *C* and, most importantly, the exponent *n*, which is mainly determined by the pDOS and the lowest CF energy gap.^29^

In the high-temperature range, excited doublets of the *J* = 15/2 multiplet start to be populated. Hence, in this regime we adopt a master equation approach, accounting for all the possible transitions.^41,42^ Calculations show that relaxation is here governed by a single rate following an Arrhenius-like behavior τ_Orbach_^–1^ ≃ τ_0_^–1^*e*^–*U*_eff_/*K*_*B*_*T*^, where the coefficient τ_0_ and the effective barrier *U*_eff_ are directly evaluated with our approach. More details are given in the Supporting Information.

Before applying our approach to relaxation, we have investigated the pDOS ρ(*E*) and low-temperature CF excitations in molecular crystals of **2** and **3** with the thermal-neutrons spectrometer MERLIN at ISIS^43^ (see the Supporting Information for more details). As expected, a single magnetic excitation was detected for compound **2** at ≃62 meV, which is in agreement with the ab initio calculations (52 meV). The observed wave-vector transfer (*Q*)-dependence of the measured intensity clearly identifies the peak at 62 meV as the only magnetic excitation (see Figure S13 in the Supporting Information). A similar energy gap between the two lowest Kramers doublets was also predicted for **3** (54 meV) but was not detectable, being covered by phonon modes around 60 meV.^44^ In Figure 2a and b, we report the neutron-weighted pDOS (nwDOS) measured on **2** and **3**, respectively. To reproduce these INS data, we performed DFT (PBE) simulations to calculate the atom-projected DOS and reconstruct the nwDOS by applying the one-phonon incoherent approximation (see the Supporting Information for more details). The comparison reported in Figure 2a and b shows an excellent agreement between our calculations and the INS data for **2** and **3**, demonstrating the reliability of the calculated pDOS. The pDOS entering the model for relaxation is the one without the weighting for the neutron cross-section and is reported in Figure 2c for **2** and **3**, compared with the pDOS of **1** (for the pDOS over the full energy range, see Figure S11 in the Supporting Information).

**Figure 2:**
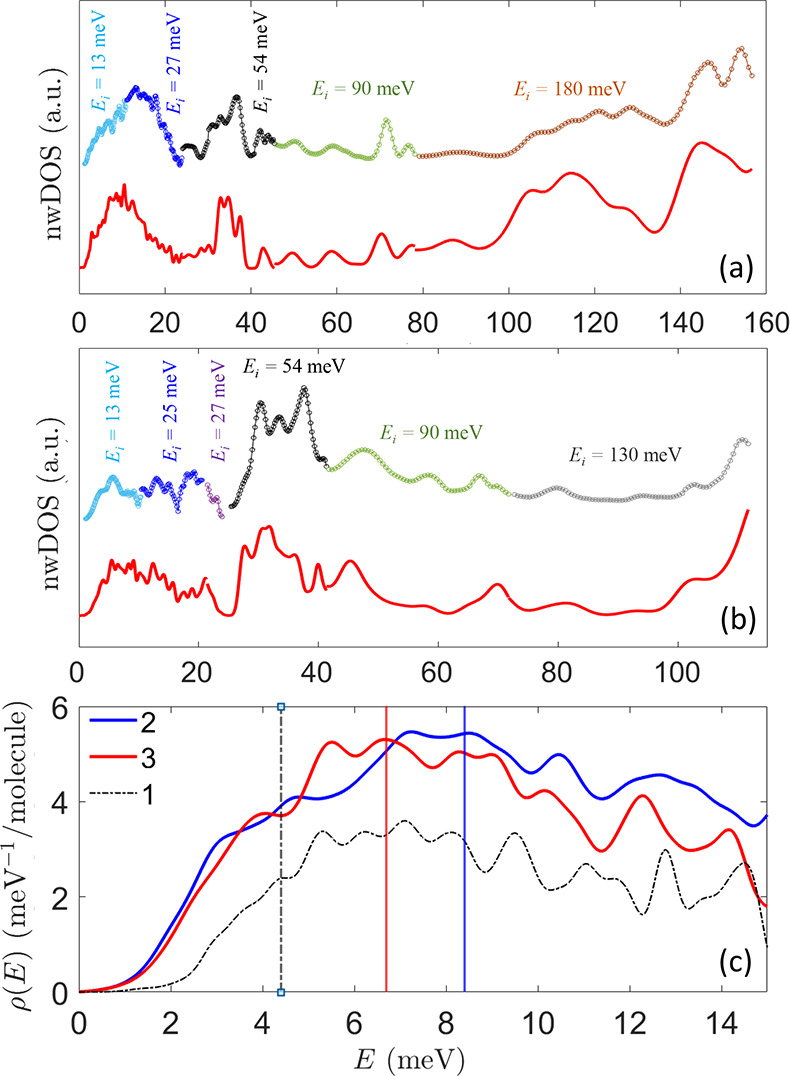
Measured (symbols) and calculated (red line) nwDOS for (a) compound **2** and (b) compound **3**. The neutron-weighted DOS was measured at *T* = 5 K with different incident neutron energies *E*_i_ and resolutions and are reported here with different colors. The fwhm of the calculated nwDOS varies accordingly to the experimental configuration. Error bars are of the order of the symbols size. (c) DFT (PBE) low-energy pDOS ρ(*E*) of compounds **2** and **3** (blue and red lines, respectively) compared to the ρ(*E*) of **1** (dashed black line) obtained with a single fwhm = 0.29 meV. For the sake of comparison, each pDOS has been normalized to the number of molecules in the unit cell. Vertical lines pinpoint the threshold energy ℏω_*D*_.

Measured relaxation rates τ^–1^ are shown in Figure 3a.^24,30^ The presence of two different regimes is evident: an intermediate-temperature Raman region and a high-temperature Arrhenius regime. Lines in Figure 3a are the result of our calculations and were obtained without any assumption on the functional form of the relaxation rate. It is evident that the different behaviors of the three compounds are well-reproduced in the whole temperature range. A comparison between calculated curves and experimental points is the best figure for the merit of our approach. Conversely, the derivation of *effective* relaxation coefficients is often not univocal because these coefficients are often strongly correlated and temperature-dependent. In our approach, Raman and Orbach mechanisms are disentangled by separately computing their respective contributions. The former, as shown in Figure 3b, exhibits a power-law behavior (with a single exponent *n*) only in a narrow temperature region, and the related coefficients strongly depend on the choice of such a range. In particular, the value *n* varies from 2 to 9 when going from the high temperature limit to the low temperature limit. Conversely, in the present systems the Orbach parameters *U*_eff_ and τ_0_ are stable and can be obtained by focusing on the high-temperature range. Fitted Hamiltonian parameters and effective relaxation coefficients determined from calculations for **1**, **2**, and **3** are summarized in Table 1. We note that the trends in the Raman exponent *n* and in the effective barrier *U*_eff_ extracted from the phenomonological fitting of experimental data (see the Supporting Information and refs (17), (24), and (30) for **1**, **2**, and **3**, respectively) are in agreement with our model. Indeed, in both cases compound **1** shows the largest *U*_eff_ value and the smallest *n* value , complex **3** is intermediate, and **2** is characterized by the smallest *U*_eff_ value and the largest *n* value.

**Table 1:** Fitted Hamiltonian Parameters and Calculated Relaxation Coefficients

fitted	**2**	**3**	**1**(29)
ℏω_*D*_ (meV)	8.6 ± 0.7	6.7 ± 0.5	4.4
ζ_*a*_ (10^–9^ s^1/2^)	4.7 ± 0.5	0.21 ± 0.03	1.8
ζ_*o*_ (s^–1/2^)	33 ± 6	13 ± 5	0.9
determined from model calculations			
*n*	3.6 ± 0.2	3.0 ± 0.1	2.3
*C* (K^–*n*^ s^–1^)	(5 ± 3) × 10^–5^	(3 ± 1) × 10^–3^	4 × 10^–7^
*U*_eff_ (K)	1093	1127	1786
τ_0_ (s)	(1.5 ± 0.5) × 10^–13^	(3 ± 2) × 10^–12^	1.8 × 10^–11^
average axiality^a^	0.866	0.588	0.966

a axiality = |⟨*m*_*J*_|ψ_*i*_⟩|^2^, where |ψ_*i*_⟩ is the CF eigenstate and |*m*_*J*_⟩ is the state with the largest component averaged over all the doublets of the *J* = 15/2 multiplet.

**Figure 3:**
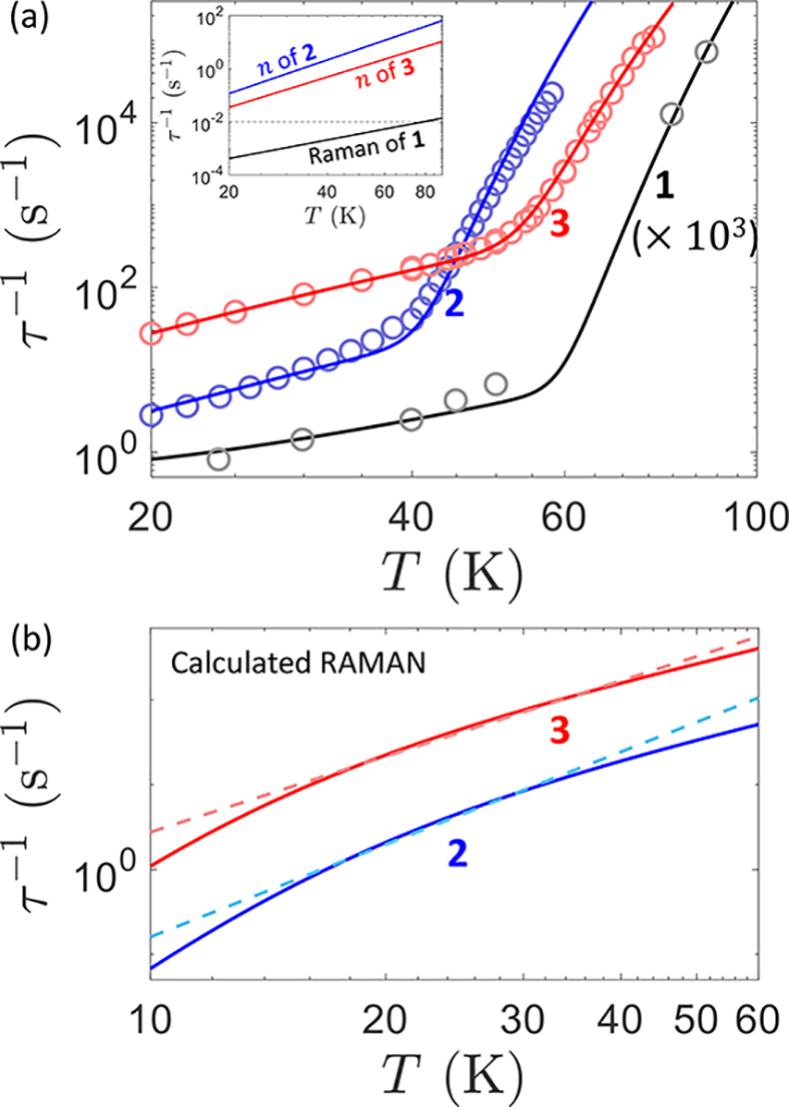
Relaxation rate τ^–1^ for **2** (blue scatters) and **3** (red scatters) compared with relaxation rates of **1** (gray scatters). Blue, red and gray lines are the full simulation of τ^–1^ comprising Raman and Orbach contributions. Inset: comparison of the relaxation rate τ^–1^ of **1** calculated with our model and different exponent *n* for the Raman power-law: original values (black line), value of **2** (blue line), value of **3** (red line). The dashed line highlights the value τ^–1^ = 10^–2^ s^–1^, for the extrapolation of T_*B*_ as the temperature at which the relaxation time is 100 s. (b) Calculated Raman relaxation rates for compound **2** and **3** (continuous lines) and corresponding fits to extract *effective* Raman coefficients *C* and *n* (dashed lines) using a power-law *T*-dependence in the 15–35 K (15–45 K) range for complex **2** (**3**).

Compound **1** has several characteristics that contribute to the suppression of Raman mechanisms (eq 2). The large CF splittings and the relatively small coupling ζ_*o*_ with nondispersive modes make optical phonons ineffective for Raman relaxation. Moreover, the pDOS slowly increasing with energy and the rather small value of ℏω_*D*_ yield a weak temperature dependence, with calculated *n* ≃ 2 in the Raman region, which is in agreement with experimental findings. Conversely, the steeper shape of the pDOS of **2** and **3** at low energies and the larger ℏω_*D*_ values lead to more efficient Raman processes with higher exponents *n* = 3.6 and 3.0, respectively. This in turn leads to a much faster relaxation in **2** and **3**, which are characterized by relaxation rates several orders of magnitude larger than that of **1** in the intermediate temperature range (τ^–1^ ∼ 10^1^–10^2^ s^–1^ vs 10^–3^ s^–1^).

Our approach also reproduces the multistep Orbach regime and yields calculated energy barriers *U*_eff_ = 1786, 1093, and 1127 K for **1**, **2**, and **3**, respectively. An analysis of CF coefficients and eigenstates shows that **1** displays the largest *average axiality* (i.e., the ground and excited CF doublets are practically characterized by a single |*m*_*J*_| component). Our calculations also demonstrate that the lower value of *U*_eff_ in **2** and **3** reflects the energy of the respective second-excited CF doublets, which are the lowest in energy to lose axiality (see Table S8 in the Supporting Information). Moreover, the larger coupling ζ_*o*_ with optical modes of **2** and **3** leads to shorter τ_0_ and faster relaxation in the Orbach regime.

Figure 3 shows that suppressing the Raman mechanism is fundamental to increase the blocking temperature of rare-earth SMMs toward the high-temperature (Orbach) range. Indeed, if the temperature dependence of the relaxation rate of **1** is recalculated by replacing the exponent *n* (depending on the pDOS and CF splittings, see above) with that of **2** or **3**, a steeper power-law for τ^–1^(*T*) and a large overall increase of the relaxation rates are obtained (see inset of Figure 3a).

Raman relaxation also strongly depends on the coupling with dispersive modes ζ_*a*_. Our calculations point to a small value of ζ_*a*_ for **3**, consistently with **3** being a neutral molecule, whereas a counterion is present in **1** and **2** (see Figure 1). Indeed, in **1** and **2** we expect stronger modulations of the CF (and a larger value for ζ_*a*_), because low-energy dispersive modes induce the motion of charged objects (ion and counterion). Despite having a much weaker coupling ζ_*a*_ with dispersive modes, **3** is characterized by a larger calculated value of *C* (see Table 1). Indeed, our calculations show that this parameter is largely affected by the average axiality of the lowest eigenstates, which is much lower for **3**. Moreover, the pDOS at low energy of **3** is steeper than in **2** (see Figure 2c) and also contributes to the larger *C* value. Thus, the CF and the form of the pDOS compete with the spin–phonon coupling ζ_*a*_ in determining the prefactor *C* for the Raman power-law.

By combining all these hints, we can now draw a recipe to guide future synthetic efforts devoted to improve the performance of single-molecule-based memories by suppressing both Raman and Orbach processes. The essential ingredients are the following:

1. A crystal-field yielding
  (a) axial ground and excited doublets, which could be achieved by increasing the axial symmetry and the ratio of axial to equatorial ligand donor strength. These ingredients are important to limit Raman relaxation (through a reduction of both *C* and *n*) as well as to increase the effective barrier *U*_eff_ by hindering short-cuts in the relaxation path.
  (b) large CF gaps, which could also be achieved by increasing the ratio of the axial to equatorial ligand donor strength. A large gap between ground and low excited doublets is important to prevent Raman-like resonant two-step processes (which would effectively increase *n* in a narrow temperature range, see ref (29)), while the overall CF splitting affects the value of *U*_eff_ in the Orbach process.
2. Shape of the pDOS at low energy, which must be engineered to limit the efficiency of Raman mechanism. This can be achieved by
  (a) making it not too steep, i.e., slowly increasing with energy (thus reducing the prefactor *C*).
  (b) displaying a rather small ℏω_*D*_ to reduce the value of *n* and approach the high-temperature limit *n* = 2.
  Both of these are complicated to control with chemistry; however, a guidance is to raise the energy of intramolecular vibrations and lower the energy of intermolecular ones.^29^ In general, although difficult to predict from structural changes, the pDOS can be computed by DFT starting from a given molecular structure.^45^
3. pDOS at energies corresponding to CF excitations, which must be as small as possible to increase τ_0_, thus quenching the resonant (Orbach) mechanism.^27^ This means reducing the number of vibrational modes at those energies, moving most of them out-of-resonance with the most likely CF transitions. This is hard to control a priori but can be reliably calculated for candidate molecular structures,^27^ so in principle designs can be tested.
4. Keeping magneto–elastic coupling with local modes close in energy to CF gaps (ζ_*o*_) as small as possible, again to increase τ_0_. This means that phonon modes at those energies must not induce a significant modulation of the Dy ligand cage. Again, this can be reliably calculated for SMM candidates.^27^
5. A small magneto–elastic coupling with dispersive modes (ζ_*a*_) to suppress the value of *C*. This could be achieved by reducing the mixing between acoustic and optical modes^45^ or by choosing neutral molecules. Indeed, it was recently shown^45^ that ζ_*a*_ is largely dominated by the optical component of the modes. This can be reduced, for instance, by moving low-lying optical modes to higher energies. Moreover, the Coulomb interaction between two close charged objects (magnetic core and counterion) can lead to a large modulation of the CF and thus to a large ζ_*a*_.

Below we summarize the effect of these different ingredients on Raman and Orbach relaxation:

1. The Raman exponent *n* is controlled by the axiality and gaps of the (lowest) CF doublets (point 1) and by the shape of the pDOS at low energy (point 2). *n* close to 2 can be achieved by axial CF doublets, large CF gaps, and a small ℏω_*D*_ values.
2. The Raman prefactor *C* is limited by fulfilling points 1, 2(a), and 5.
3. Long τ_0_ are obtained by satisfying points 3 and 4.
4. Large barriers *U*_eff_ are achieved in the presence of large CF gaps and strongly axial ground and excited doublets (point 1). Indeed, in the examined cases we find that *U*_eff_ roughly corresponds to the gap between the ground and the first nonaxial doublet, which activates through-barrier relaxation.

In conclusion, we have demonstrated the power of our effective approach for the calculation of the magnetic relaxation by applying it to three high-barrier Dy-based SMMs characterized by very different relaxations. The application of the method to these compounds pinpoints the crucial role played by the Raman mechanism in the new generation of 4f SMMs and highlights the main ingredients controlling it. This comparative analysis thus supplies new hints for the recipe to design new SMMs. In addition, this study also provides new tools for the investigation of the relaxation dynamics of other molecular systems, such as molecular qubits, whose coherence times can be influenced by phonon-induced relaxation.^45,46^

## Supporting Information Available

Synthesis of the samples, details of the neutron scattering experiment, ab initio calculations, theoretical procedures, and supplementary results (PDF)
