# Supplementary material for: A Cost-Effective Semi-Ab Initio Approach to Model Relaxation in Rare-Earth Single-Molecule Magnets
Source: J Phys Chem Lett. 2021 Sep 7;12(36):8826–32. doi: 10.1021/acs.jpclett.1c02367 (PMC8450932; doi:10.1021/acs.jpclett.1c02367)
Supplement: Supplementary file 1 — jz1c02367_si_001.pdf [file jz1c02367_si_001.pdf]

Supporting Information

A Cost-Effective Semi Ab-initio Approach to Model Relaxation in Rare-Earth Single-Molecule Magnets

Elena Garlatti,[a,b] Alessandro Chiesa,[a,b] Pietro Bonfà,[a] Emilio Macaluso,[a,b] Ifeanyi J. Onuorah,[a] Vijay S. Parmar,[c] You-Song Ding,[d] Yan-Zhen Zheng,[d] Marcus Giansiracusa,[c] Daniel Reta,[c*] Eva Pavarini,[e] Tatiana Guidi,[f] David P. Mills,[c] Nicholas F. Chilton,[c] Richard E. P. Winpenny,[c] Paolo Santini,[a,b] Stefano Carretta,[a,b]

[a] Università di Parma, Dipartimento di Scienze Matematiche, Fisiche e Informatiche, 43124 Parma, Italy;
[b] UdR Parma, INSTM, I-43124 Parma, Italy.
[c] Department of Chemistry, The University of Manchester, Oxford Road, Manchester, M13 9PL, UK.
[d] Frontier Institute of Science and Technology, Xi'an Jiaotong University, 99 Yanxiang Road, 710054 Xi'an, Shaanxi, China.
[e] Institut for Advanced Simulation Forschungszentrum Juelich and JARA High-Performance Computing, RWTH Aachen University, 52062 Aachen, Germany.
[f] ISIS Facility, Rutherford Appleton Laboratory, Didcot OX11 0QX, UK.
*Present Address: Department of Inorganic and Organic Chemistry, University of Barcelona, Barcelona, Spain.

Synthesis

General experimental procedures

All syntheses and manipulations were conducted under argon with the rigorous exclusion of oxygen and water using Schlenk line and glovebox techniques. THF was obtained from a central solvent purification facility (SPS drying columns), dried further over 4 Å molecular sieves for at least 1 week, and degassed before use. For NMR spectroscopy C_6D_6 was dried by refluxing over potassium, and was vacuum transferred and degassed by three freeze-pump-thaw cycles before use. [Ln(^{t}BuO)Cl(THF)$_5$][BPh_4]·2THF (Ln = Dy, **2**; Y, **2-Y**),[1] [Dy(Mes*O)$_2$(THF)$_2$Br] (**3**)[2], Mes*OK[3] and anhydrous YBr_3[4] were prepared according to published procedures. ^{1}H (400 MHz) and ^{13}C{^{1}H} (100 MHz and 125 MHz) NMR spectra were obtained on an Avance III 400 MHz or 500 MHz spectrometers at 298 K. These were referenced to the solvent used, or to external TMS (^{1}H, ^{13}C). ATR-IR spectra were recorded as microcrystalline powders using a Bruker Tensor 27 ATR-Fourier Transform infrared (ATR-FTIR) spectrometer. Elemental analyses were performed by Mrs Anne Davies and Mr Martin Jennings at the Microanalysis Service in the Department of Chemistry at The University of Manchester, UK.

*[Y(Mes*O)$_2$(THF)$_2$Br]·3THF (3-Y·3THF):*

A mixture of YBr_3 (0.326 g; 1 mmol) and two equivalents of Mes*OK (0.605 g; 2 mmol) in a Schlenk flask was cooled to -78 ^{o}C before adding 30 ml of THF. The reaction mixture was refluxed with continuous stirring for 1.5 hours, then stirred at room temperature for further 16 hours. After the stirring was stopped, the cloudy reaction mixture settled into a grey powder (KBr) and clear, colorless supernatant (product). The supernatant was filtered and concentrated to get colorless block-shaped crystals of **3-Y**·3THF (0.485 g, 0.46 mmol; 46%) when stored for 36 hours at 0 ^{o}C. The crystals were dried in vacuo to afford a white crystalline solid, which ^{1}H NMR spectroscopy indicates is desolvated **3-Y**. Anal. Calcd for $C_{44}H_{74}BrO_4Y$: C, 63.22; H, 8.92 Found (%): C, 61.90; H, 9.02. Elemental analysis results show lower carbon values than predicted, which we attribute to carbide formation from incomplete combustion. ^{1}H NMR (400.07 MHz, C_6D_6, 298 K, ppm): δ = 0.95, (m, 8H, THF-OCH$_2$C*H*$_2$), 1.42 (s, 18H, *p*-C(CH$_3$)$_3$), 1.75 (s, 36H, o-C(CH$_3$)$_3$), 3.84 (m, 8H, THF-OC*H*$_2$CH$_2$), 7.59 (s, 4H, *m*-CH) (see Figure S1). ^{13}C{^{1}H} NMR (400.07 MHz, C_6D_6, 298 K, ppm): δ = 25.41 (THF-OCH$_2$CH$_2$), 32.73 (o-C(*C*H$_3$)$_3$), 33.20 (p-C(*C*H$_3$)$_3$), 35.07 (p-*C*(CH$_3$)$_3$), 36.45 (o-C(*C*H$_3$)$_3$), 72.98 (THF-OCH$_2$*C*H$_2$), 122.66 (m-Ar), 138.24 (o-Ar), 138.84 (p-Ar), ipso-Ar not observed (see Figure S2). FTIR (ATR, microcrystalline): ῦ = 2949 (m, br), 2904 (w), 2871 (m, br), 1475 (w), 1457 (w), 1416 (w), 1385 (w), 1356 (w), 1231 (s), 1200 (m), 1153 (w), 1120 (s), 1009 (s), 921 (w), 889 (m, br), 877 (m, br), 857 (m, br), 832 (m, br), 817 (m, br), 780 (s), 746 (s), 672 (w), 643 (w), 532 (m), 456 (m), 435 (m) cm–1 (see Figure S3).

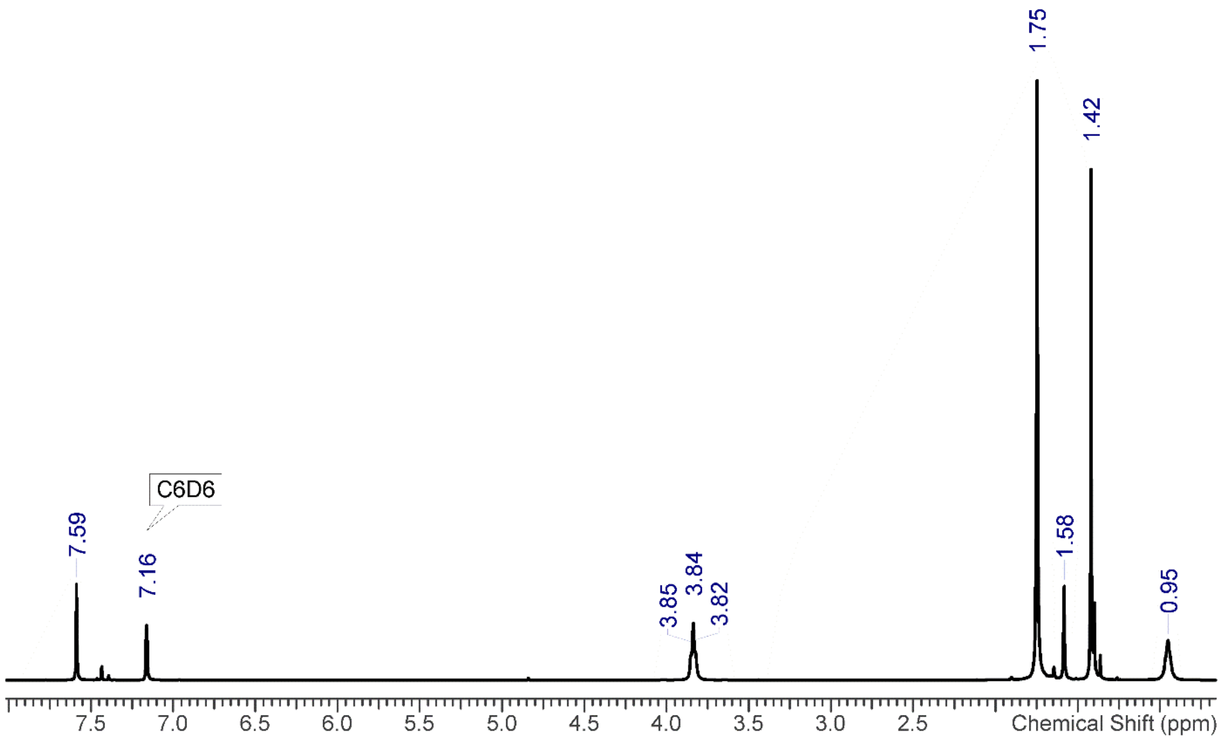

FIG. S1. ^{1}H NMR spectrum of [Y(Mes*O)$_2$(THF)$_2$Br] (**3-Y**) in C_6D_6.

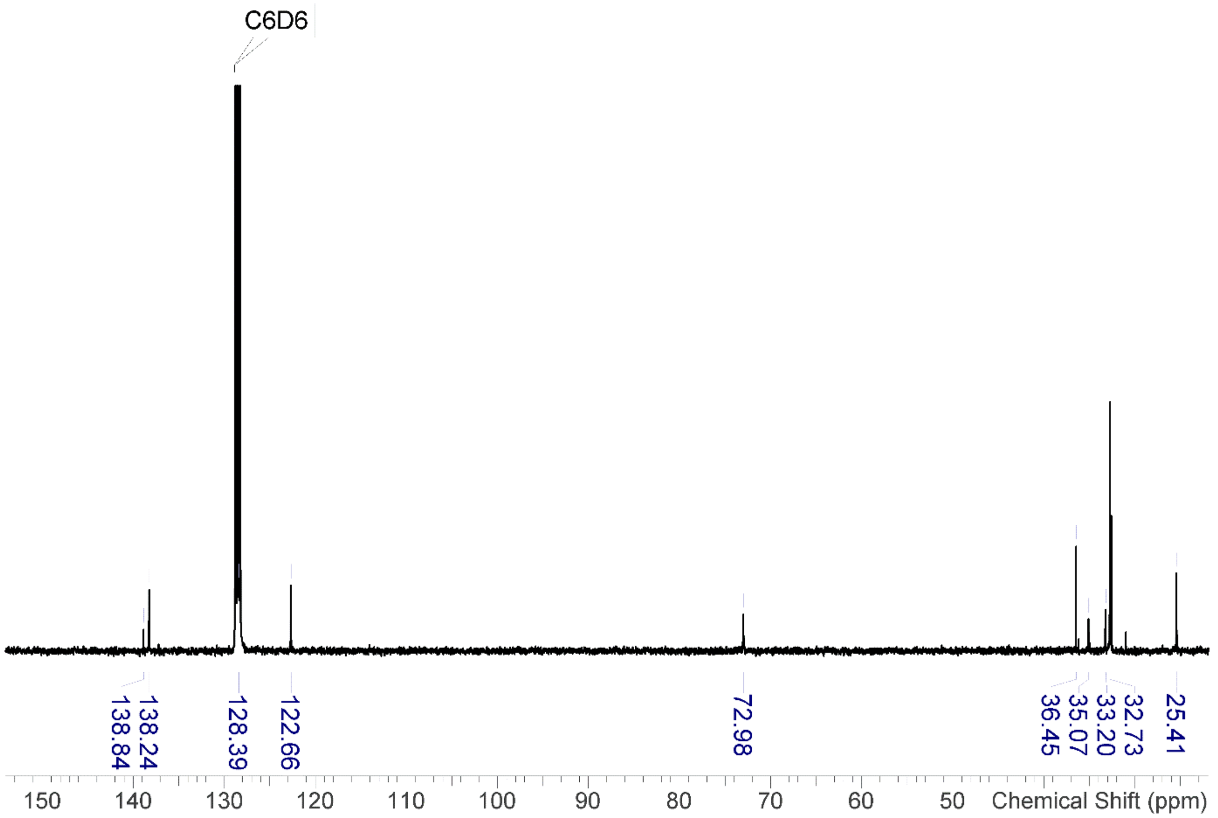

FIG. S2. ^{13}C NMR NMR spectrum of [Y(Mes*O)$_2$(THF)$_2$Br] (**3-Y**) in C_6D_6.

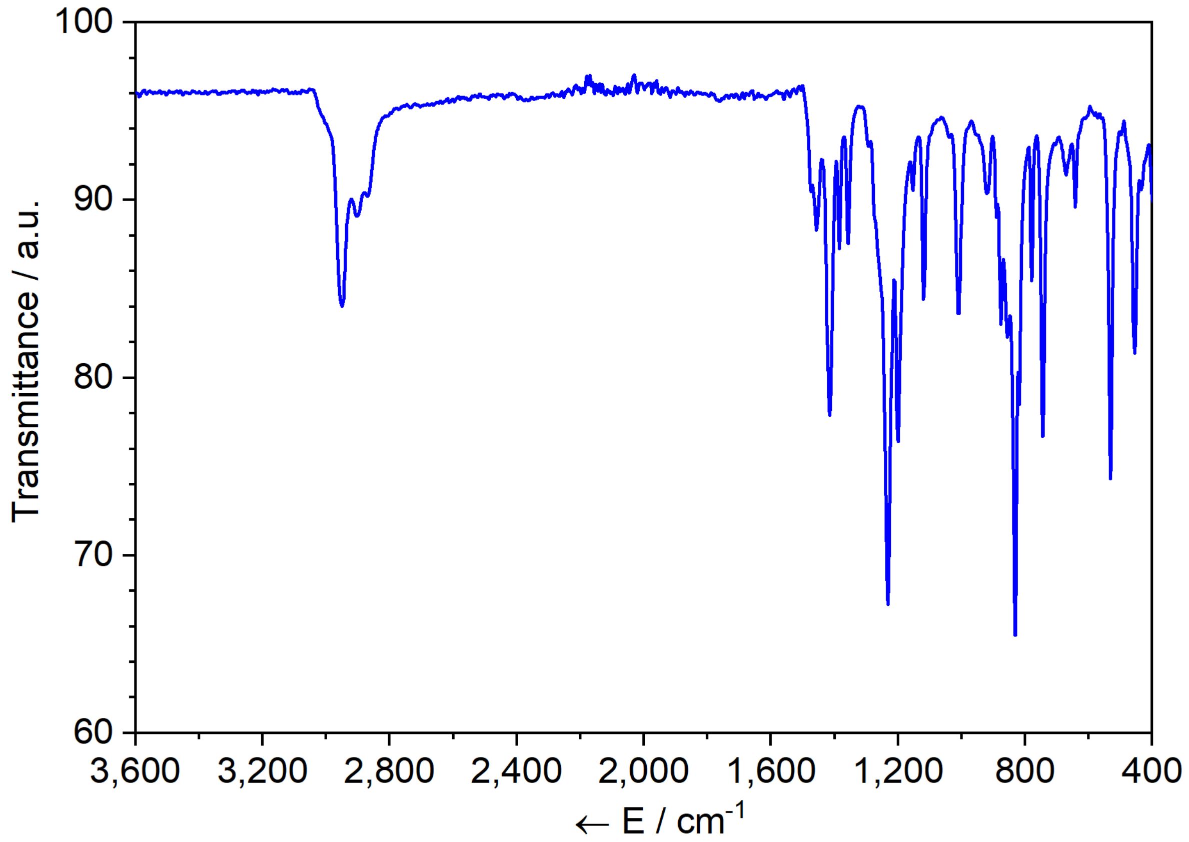

FIG. S3. ATR-IR spectrum of $[Y(Mes^*O)_2(THF)_2Br]\cdot 3THF$ (**3-Y**), 400-3600 cm^{-1}.

Crystallography

Single crystal XRD was performed on crystalline batches of **2**,[2] **2-Y**[2] **3**[2] and **3-Y**. For all complexes apart from **3-Y** we were able to confirm the identity of these samples by the close agreement of unit cell parameters to those reported previously in the literature (See CCDC 1450752, 1566471 and 1978054).

The crystal data for [Y(Mes*O)$_2$(THF)$_2$Br]·3THF is compiled in Table S1. The crystals were examined using an Oxford Diffraction Supernova diffractometer, equipped with CCD area detector and mirror-monochromated Mo Kα radiation (λ = 0.71073 Å). Intensities were integrated from data recorded on 1° frames by ω rotation. Cell parameters were refined from the observed positions of all strong reflections in each data set. A multi-scan absorption correction with a beam profile was applied.[5] The structures were solved using direct methods by SHELXS; the datasets were refined by full-matrix least-squares on all unique F^2 values.[6] CrysAlisPro[5] was used for control and integration, and SHELX[6,7] was employed through OLEX2[8] for structure solution and refinement. ORTEP-3[9] and POV-Ray[10] were employed for molecular graphics. CCDC #2088557 contains the supplementary crystal data for this article. These data can be obtained free of charge from the Cambridge Crystallographic Data Centre via www.ccdc.cam.ac.uk/data_request/cif.

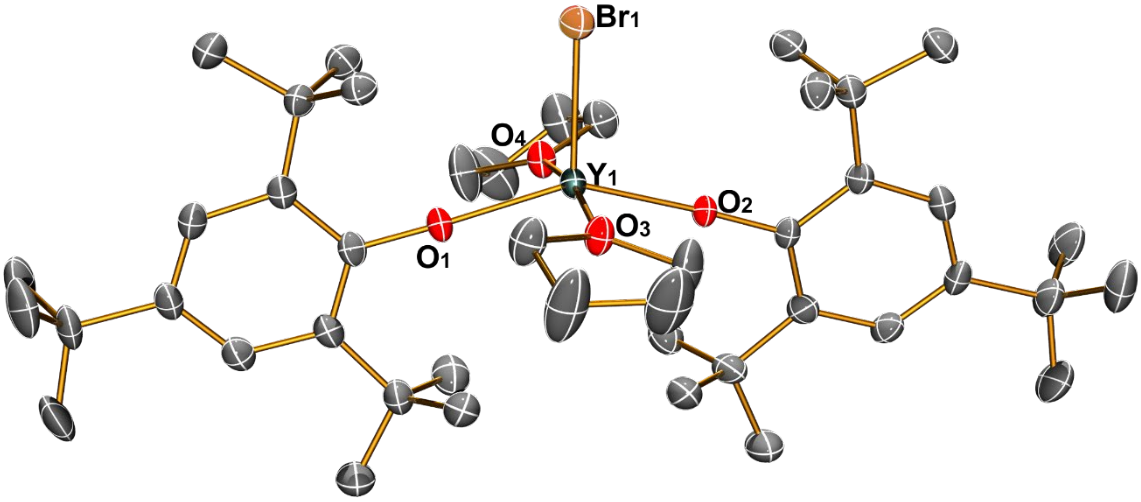

FIG. S4: View of the molecular structure of [Y(Mes*O)$_2$(THF)$_2$Br]·3THF (**3-Y**·3THF) from single crystal XRD at 150 K with thermal ellipsoids drawn at 40% probability level (Dy turquoise, Br brown, O red, C grey). H atoms and lattice solvent are omitted for clarity.

TAB. S1. Crystallographic data for [Y(Mes*O)$_2$(THF)$_2$Br]·3THF (**3-Y**·3THF).

	[Y(Mes*O)$_2$(THF)$_2$Br]·3THF
Formula	$C_{56}H_{98}BrO_7Y$
Fw, g mol^{-1}	1052.16
Cryst size, mm	0.262 x 0.178 x 0.152
Crystal system	monoclinic
Space group	$P2_1/c$
Collection Temperature (K)	150(2)
a, (Å)	17.6623(9)
b, (Å)	15.9491(7)
c, (Å)	20.6765(11)
α, (°)	90
β, (°)	102.179(5)
γ, (°)	90
V, (Å^3)	5693.4(5)
Z	4
ρ_{calc} g cm^{-3}	1.227
μ, mm^{-1}	1.773
No. of reflections measured	22025
No. of unique reflections, R_{int}	10424, 0.0485
No. of reflections with $F^2 > 2s(F^2)$	6776
Transmission coefficient range	0.538-1.000
R_1 $(I > 2\sigma(I))$	0.0535, 0.1021
wR_2 (all data)	0.1032, 0.1208
S^a	1.018
Parameters, Restraints	696, 948

Max./min. difference map, e Å^{-3}	0.628, -0.446

^{a}Conventional $R = \Sigma||F_o| - |F_c||/\Sigma|F_o|$; $R_w = [\Sigma w(F_o^2 - F_c^2)^2/\Sigma w(F_o^2)^2]^{1/2}$; $S = [\Sigma w(F_o^2 - F_c^2)^2/\text{no. data} - \text{no. params})]^{1/2}$ for all data.

TAB. S2: Selected bond lengths (Å) and angles (°) in [Y(Mes*O)$_2$(THF)$_2$Br]·3THF (**3-Y**·3THF).

**Lengths**	**3-Y**	**Angles**	**3-Y**
Y—O1	2.109(3)	O1-Y-O2	146.4(1)
Y—O2	2.110(2)	O1-Y-O3	86.33(9)
Y—O3	2.348(3)	O1-Y-O4	87.94(9)
Y—O4	2.325(2)	O2-Y-O3	88.89(9)
Y—Br	2.6876(6)	O2-Y-O4	90.62(9)
		O3-Y-O4	168.85(9)
		Br-Y-O1	108.74(7)
		Br-Y-O2	104.62(7)
		Br-Y-O3	92.23(7)
		Br-Y-O4	98.67(7)

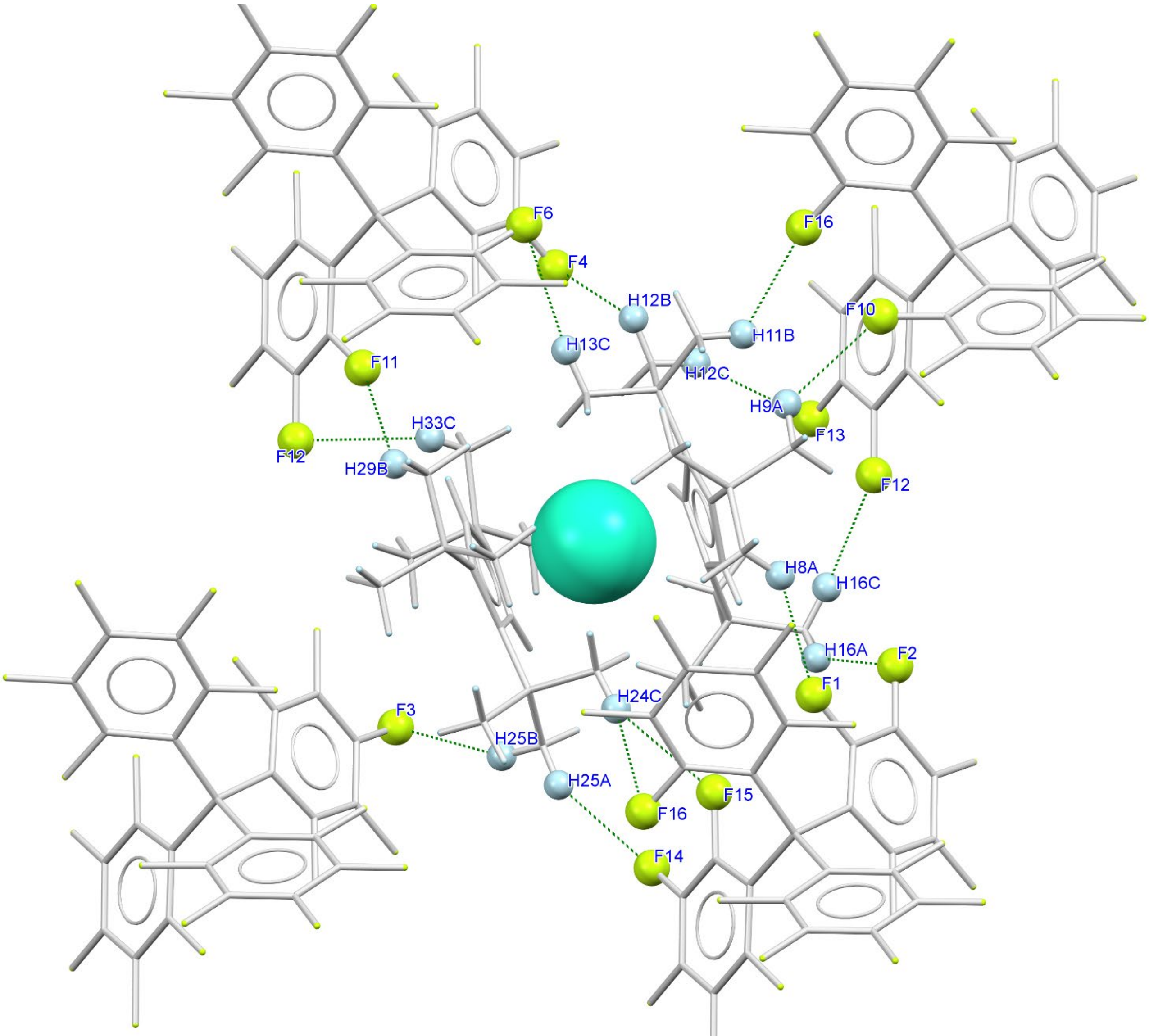

FIG. S5: View of the packing of the $[B(C_6F_5)_4]$ anion and the dysprosocenium cation held by the hydrogen bonding network in $[Dy(C_5H_2{}^tBu_3\text{-}1,2,4)_2][B(C_6F_5)_4]$. Colors (Dy teal, F lime, C light grey, H light blue); only the atoms showing short contacts and the metals are shown in Ball and Stick mode for clarity.

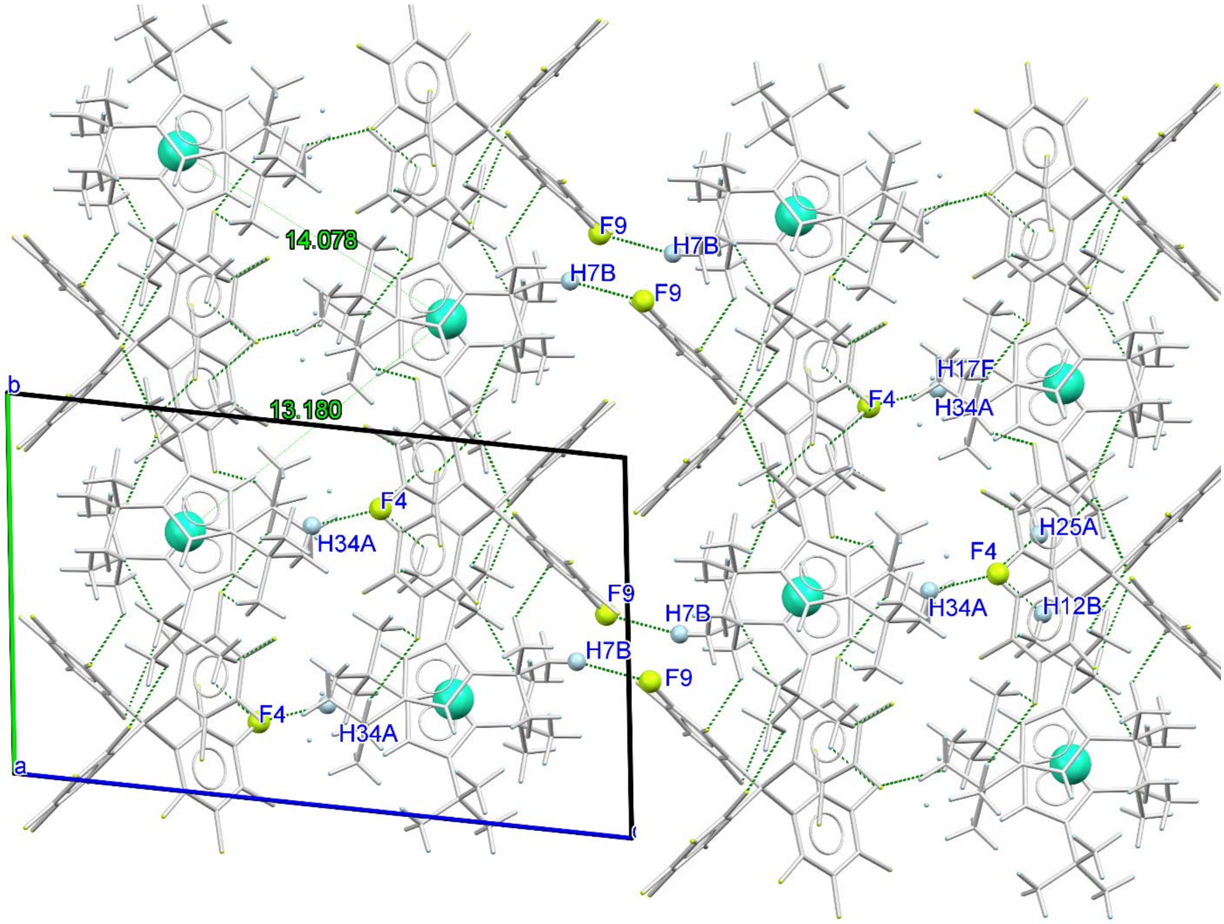

FIG. S6: View of the hydrogen bonding network between two layers in the crystal packing along the x-axis in $[Dy(C_5H_2{}^tBu_3\text{-}1,2,4)_2][B(C_6F_5)_4]$ (**1**). Colors (Dy teal, F lime, C light grey, H light blue); only the atoms showing short contacts and the metals are shown in Ball and Stick mode for clarity.

TAB. S3. The F···H short contact distances (Å) and angles (°) in $[Dy(C_5H_2{}^tBu_3\text{-}1,2,4)_2][B(C_6F_5)_4]$ (**1**).

Atom1	Atom2	Distance	C-F···H angle	F···H-C angle
F1	H8A	2.532	156.1	137.0
F2	H16A	2.240	108.8	140.2
F3	H25B	2.638	90.2	154.3
F4	H12B	2.656	110.2	124.9
F4	H34A	2.450	150.2	122.1
F9	H7BD	2.574	119.1	166.1
F10	H9A	2.730	137.6	108.5
F11	H29B	2.707	160.2	139.9
F12	H32K	2.757	136.8	140.7
F12	H33C	2.726	84.8	72.0
F12	H16C	2.704	141.5	158.1
F13	H12C	2.717	87.5	154.4
F13	H32U	2.746	126.4	163.6
F14	H25A	2.703	108.1	123.6
F15	H24C	2.727	125.4	129.4

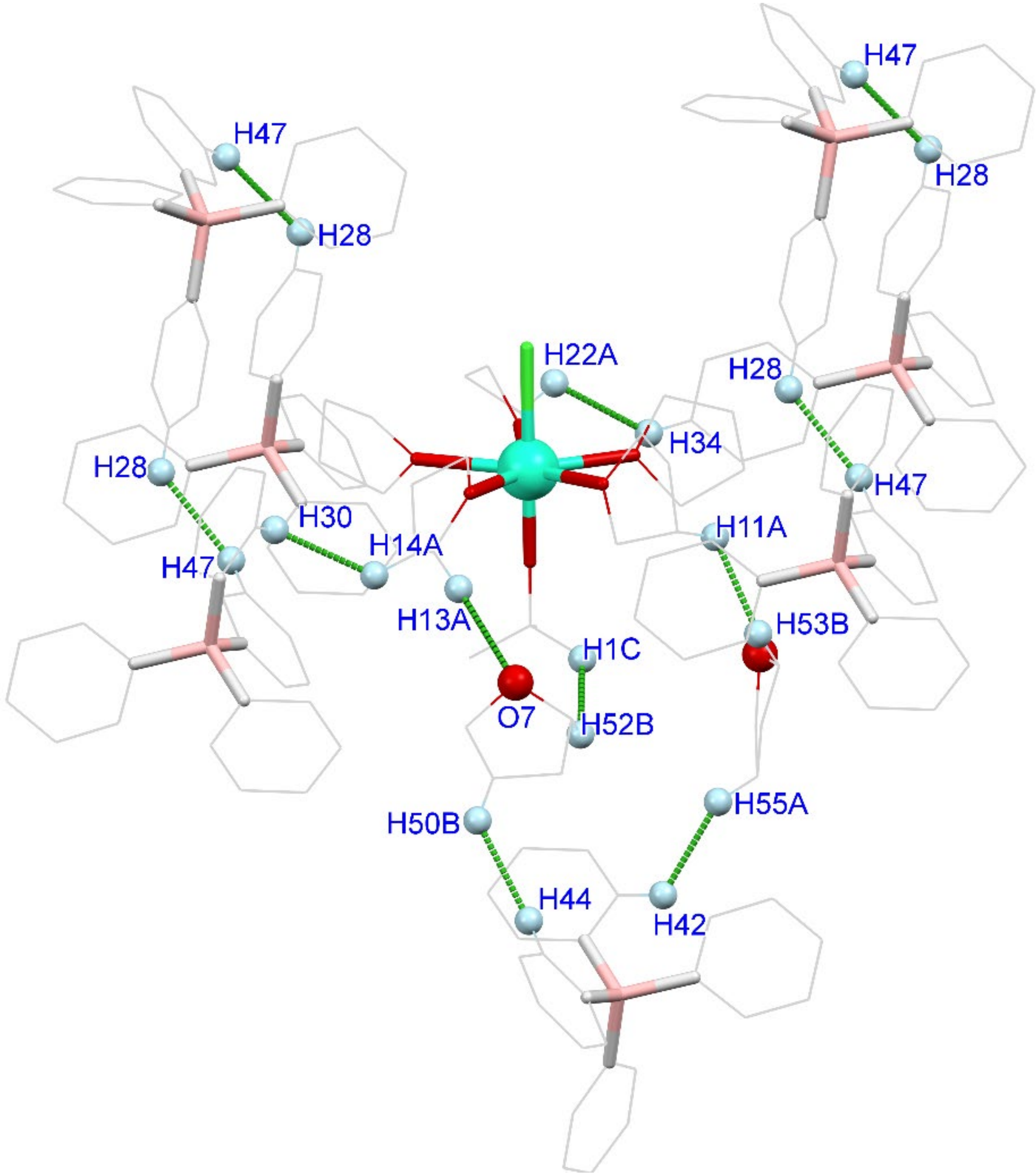

FIG. S7. View of the packing of the $[B(C_6H_5)_4]$ anion and the $[Dy(^tBuO)Cl(THF)_5]$ cation held by the hydrogen bonding network in $[Dy(^tBuO)Cl(THF)_5][BPh_4]\cdot 2THF$ (**2**). Colors (Dy teal, B pink, O red, Cl green, C light grey, H light blue); only the atoms showing short contacts are shown in Ball and Stick mode for clarity.

TAB. S4. The H···O and H···H short contact distances (Å) in $[Dy(^tBuO)Cl(THF)_5]$ $[B(C_6H_5)_4]$ (**2**).

**Atom1**	**Atom2**	**Length**
H13A	O7	2.692
H14A	H30	2.381
H22A	H34	2.371
H1C	H52B	2.389
H11A	H53B	2.244
H47	H28	2.379
H50B	H44	2.391
H55A	H42	2.325

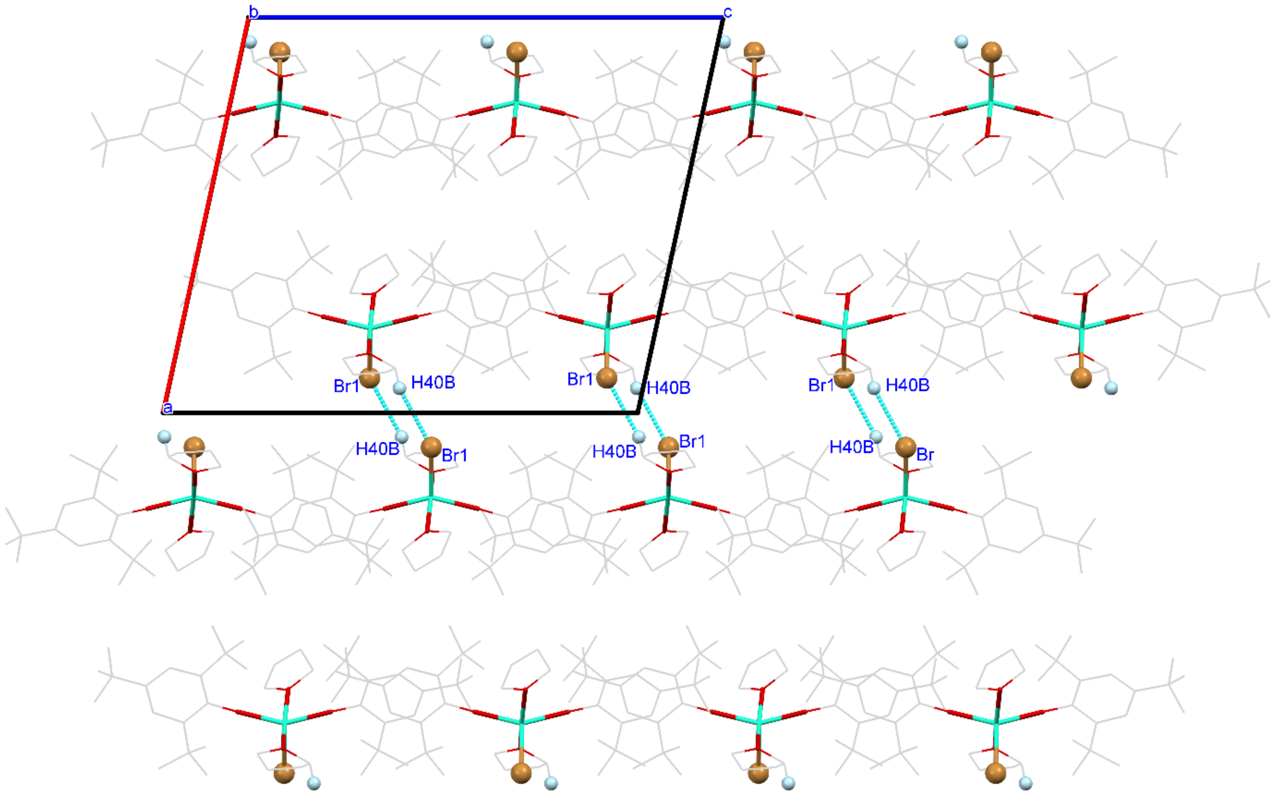

FIG. S8. View of the hydrogen bonding network between two layers in the crystal packing along the x-axis in $[Dy(Mes^{*}O)_2(THF)_2Br]$ (**3**). The Dy-$Br_1 \cdots H_{40B}$ distance is 2.938 Å. Colors (Dy teal, O red, Br brown, C light grey, H light blue); only the atoms showing short contacts are shown in Ball and Stick mode for clarity.

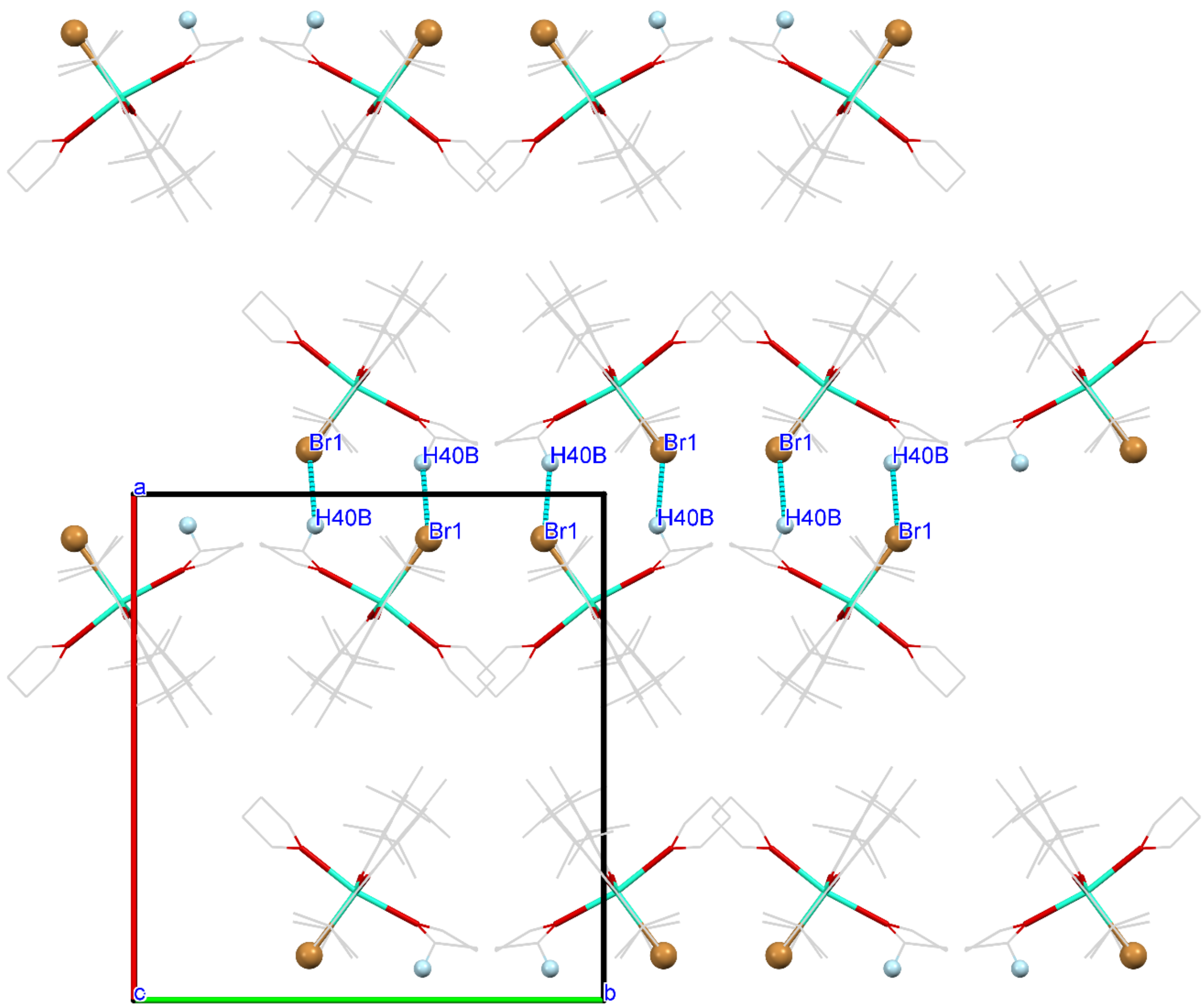

FIG. S9. View of the hydrogen bonding network between two layers in the crystal packing along the y-axis in [Dy(Mes*O)$_2$(THF)$_2$Br] (**3**). The Dy-Br$_1$···H$_{40B}$ distance is 2.938 Å. Colors (Dy teal, O red, Br brown, C light grey, H light blue); only the atoms showing short contacts are shown in Ball and Stick mode for clarity.

Ab initio calculations

Density Functional Theory (DFT) calculations

The simulations were performed with the GPU enabled version of the DFT-based code QuantumESPRESSO.[11] Norm conserving pseudopotentials were used for all atomic species[12] and the functional proposed by Perdew, Burke, and Ernzerhof (PBE)[13] was adopted for evaluating the exchange-correlation contribution. Reciprocal space was sampled with a 2x2x2 K Monkhorst-Pack grid and the cut-off energy for the expansion of the Kohn-Sham wavefunction into plane waves was set to 120 Ry. For each structure atomic positions were optimized until the residual force acting on all atomic coordinates was smaller than 1e-6 Ry/Bohr. Phonon density of states were computed using the finite displacement method as implemented in the PHONOPY package[14,15]. Atomic displacements of 0.01 Ang. were employed to obtain the force constants using 2x2x1 supercells (SC). In this latter case the plane wave cut-off was reduced to 100 Ry and reciprocal space was sampled using the gamma point only.
The phonon frequencies are reported in Fig. S10. A very small residual imaginary component is only present near the gamma point. The convergence with respect to the SC expansion was checked against the results obtained for the smaller 1x1x1 SC. We found that low energy harmonic phonon frequencies vary with the supercell size only with differences generally smaller than 0.5 meV (the only exception being a transverse mode of **2** that is correctly described only when adopting the bigger SC). We therefore concluded that the results obtained with the 2x2x1 supercells are well converged on the energy scale required for the description of the dispersive modes.
Inter-molecular hydrogen bond interactions were included in the DFT calculations. These interactions affect the shape of the phonon DOS at low energy, which, in turn has influences Raman relaxation.
The phonon density of states (pDOS) projected on the various atomic species was used to produce the neutron weighted DOS that is later compared with the experiment. Brillouin zone integration was performed using a Gaussian smearing with a variable FWHM for different energy regions (see Fig.2 in the main text). An extended pDOS for compounds **2** and **3** is reported in Fig.S11.

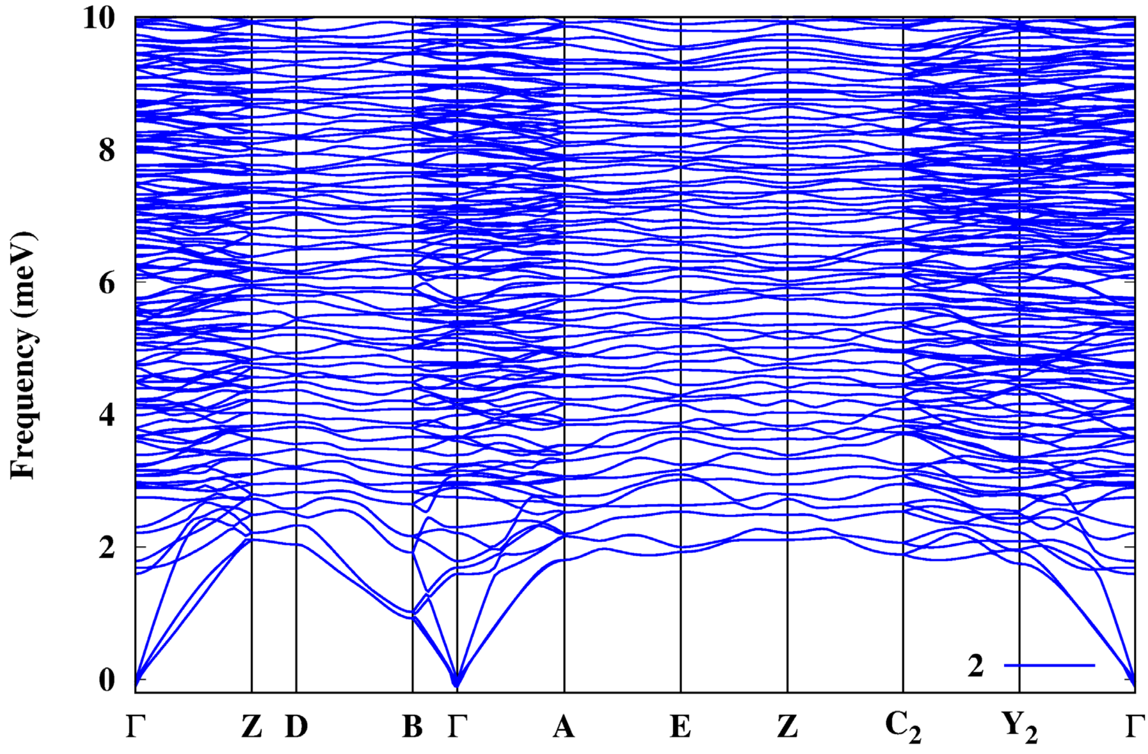

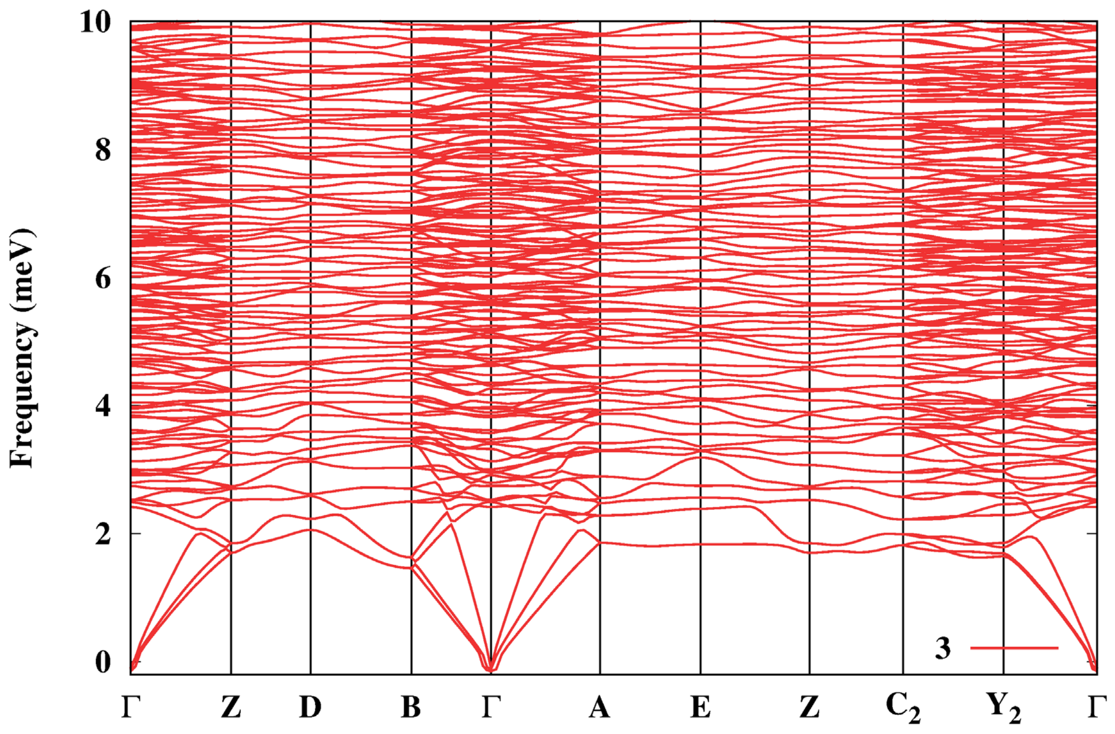

FIG. S10. DFT (PBE) low-energy phonon dispersions of compounds **2** and **3** (blue and red lines respectively).

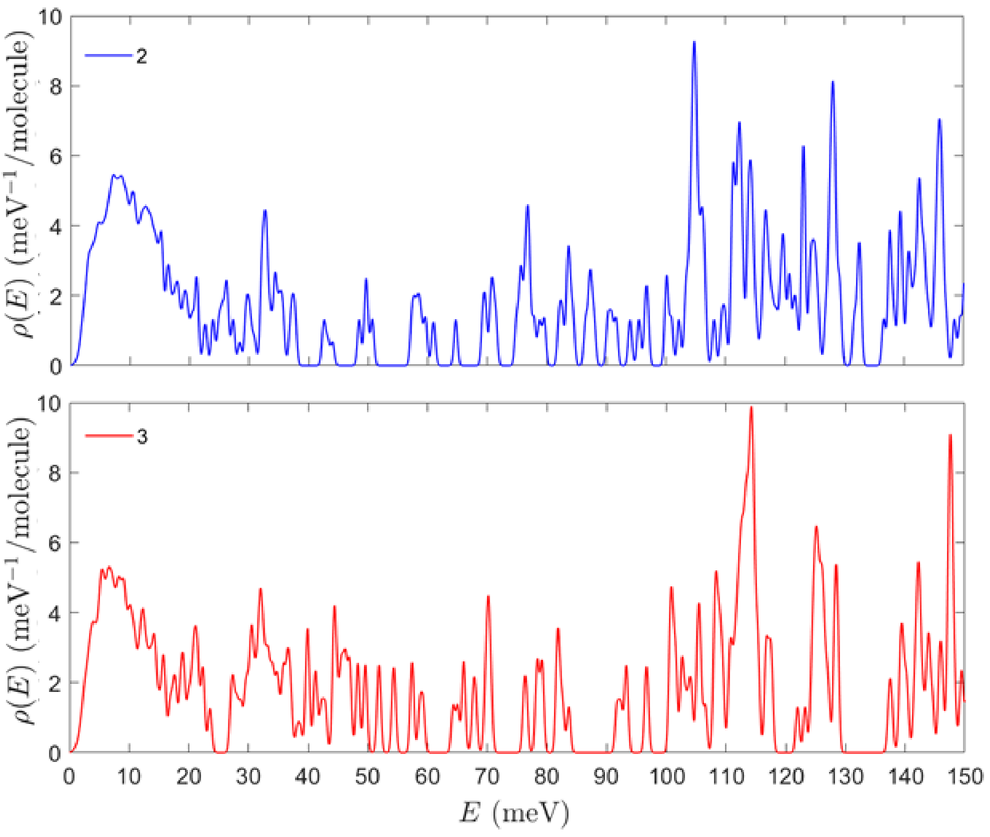

FIG. S11a. DFT (PBE) pDOS ρ(E) of compounds **2** and **3** (blue and red line respectively) up to 150 meV, obtained with a single FWHM = 0.29 meV. For the sake of comparison, each ρ(E) has been normalized for the number of molecule in the unit cell.

Gas-phase calculations

Gas-phase molecular geometry optimizations on molecules **2** and **3**, using the the XRD single crystal structures as an initial guess, were performed with Gaussian09d[16] suite of programs using the PBE[13] functional (same as for periodic calculations) with cc-pVTZ[17] basis set for all coordinating atoms, cc-pVDZ[17] for the rest of non-metal atoms, the Stuttgart RSC 1997[18] effective core potential (ECP) for the 28 core electrons of yttrium and the corresponding valence basis set for the remaining valence electrons, and Grimme's dispersion corrections.[19] To facilitate convergence, dysprosium is substituted by yttrium (where the isotopic mass is set to 162.5, that of the naturally abundant dysprosium), which is justified by their similar ionic radii and the fact that these derivatives are widely found to be structural analogues. Calculation of normal modes was performed by explicit calculation of the Hessian at the optimized geometry, making sure that the forces and displacements are zero and that all frequencies are positive. Normal modes energies of compounds 2 and 3 can be found in Table S5.

TAB. S5. Gas-phase DFT mode energies.

**2**	**3**
Frequency (meV)	Frequency (meV)
2.5778	1.4779
4.3213	2.2067
4.5901	3.3208
4.7696	3.7036
4.9871	3.7582
5.1756	3.9818
5.6695	4.4587
6.0333	4.8848
7.0825	5.3234
7.6410	6.0185
7.8842	6.2163
8.4048	6.8420
8.7229	7.2144
9.2519	7.5142
9.5589	8.7889
11.0001	9.2170
12.0302	9.5513
12.2059	10.4728
12.7451	10.5827
12.9557	11.4256
13.1385	12.0583
13.5856	12.6456
14.3497	12.7292
14.5833	13.9033
14.9752	14.0927
15.8652	14.7930
16.1981	15.2137
16.9718	15.4787
17.3490	16.1355
18.0340	16.5855
18.2837	17.0739
18.5709	18.2515

19.9368	18.5749
20.3522	18.9103
20.4337	18.9658
22.7679	19.5456
23.2960	20.5473
25.4654	20.8972
26.4475	21.4028
27.9854	22.7513
28.4044	23.2537
31.8318	24.1563
33.0047	27.8530
33.2324	28.2383
33.4793	28.4062
33.8344	28.7943
34.2873	28.8239
34.4567	30.1058
36.1901	30.5872
42.4148	30.8612
43.0690	31.2059
57.9973	31.5754
58.2356	31.8932
60.4552	32.5687
69.0146	33.0094
69.0548	33.2573
69.1176	33.3149
69.1825	33.5940
69.4657	34.0759
82.0323	35.6204
82.0643	35.7291
82.1949	36.1138
82.3297	36.3723
82.5328	36.5053
95.1295	36.9648
101.6736	37.1163
102.2115	38.9824
102.3156	39.5640
102.6099	40.1568
102.9215	40.2953
104.3358	41.6501
104.4569	41.7099
104.5115	42.5559
104.6943	43.1236
104.8792	44.0245
105.6096	44.6377
105.6860	44.8192
106.5040	44.9332
106.5389	45.0826
108.2812	45.2910
108.6505	46.5455

108.8381	46.5910
110.5562	46.6238
110.7978	46.6934
110.9129	48.6466
110.9911	48.7689
111.1484	50.0873
112.2602	50.2384
114.3101	52.1142
114.3491	52.2103
114.4022	54.7976
114.4302	54.9791
114.5546	57.0742
116.5979	57.1620
116.6756	58.3585
116.7109	58.4305
116.9575	64.7508
117.0636	65.1335
121.5273	65.8291
123.0593	65.9306
123.1244	68.0007
124.6165	68.0950
124.6458	69.5129
124.7402	72.0625
124.8629	75.7144
125.0993	75.8411
126.8785	77.6557
126.9237	77.8895
127.0348	78.5645
127.1889	78.6137
127.4936	82.6912
137.8906	84.5453
137.9249	92.2727
138.2338	92.6993
138.3731	93.6267
138.4669	93.6905
140.7607	95.8832
140.9099	95.9282
141.0059	96.7241
141.0589	101.2702
141.1922	101.3456
142.5568	101.8815
142.7083	102.9872
142.7472	103.7543
142.7683	105.3613
142.8183	105.5119
144.1783	106.0328
148.9996	107.1838
149.1480	107.2738
149.1870	107.8404

149.2669	108.3157
149.4433	108.4828
149.5354	109.1591
149.6020	109.3030
150.6211	111.4563
150.8357	111.6953
150.9797	111.7779
151.1360	112.0138
151.2190	112.2067
155.8852	112.2570
155.9495	112.2917
155.9942	112.4915
156.0284	112.7562
156.1331	112.8298
160.5607	112.8809
160.5811	112.9463
160.6109	113.0084
160.6226	113.0183
160.8297	113.1165
162.1520	113.3286
162.3655	113.3479
162.5790	113.4734
162.5820	113.5010
162.9191	113.8042
163.5623	113.8240
163.6761	113.8933
164.8481	114.4746
164.9588	114.9678
165.0556	116.3361
165.1167	117.1024
165.1265	123.4348
166.6794	123.5224
171.9091	123.5275
172.8107	123.5758
173.5107	123.7133
174.2129	123.8028
174.2628	123.8300
174.2874	123.8570
174.3227	123.9765
174.3625	124.2807
175.2280	124.3000
175.3233	124.3232
175.4450	124.3387
175.5355	124.4355
175.5858	127.3306
175.6138	128.5051
175.7251	135.9862
177.1837	136.3106
177.5033	136.5780

177.6058	138.1962
178.1030	140.8707
178.7148	140.9391
179.1094	141.5717
179.7651	143.5729
180.1906	143.8135
180.3278	144.9657
180.4402	145.0287
180.5643	146.3281
367.0851	146.8202
367.1556	146.8439
367.4237	147.0344
367.5782	147.1049
367.8820	147.1496
367.9466	147.2047
368.1906	147.2822
368.3239	147.3812
368.5595	147.5435
368.7582	147.6191
368.8099	148.2786
370.0085	148.5125
370.3687	148.6379
370.3924	149.7620
370.4077	151.4084
370.5076	151.4953
370.8678	151.5385
371.0965	152.5696
371.0982	152.9844
371.1245	153.2993
371.1893	153.3577
371.2784	154.8292
371.3712	156.1153
377.6034	156.2753
377.6392	158.2423
377.8878	158.4918
377.9584	158.5945
378.1612	160.6707
378.1776	162.4856
378.3819	162.5281
378.4409	162.6784
378.6501	162.8991
378.9598	163.0139
379.0617	163.4414
379.1391	163.4795
379.2450	163.5295
379.3621	163.9215
379.4552	164.0814
379.4896	164.1104
379.5293	164.1708

379.6392	164.1821
379.7083	164.1963
379.9263	164.5877
380.0355	164.6359
380.2558	165.2122
380.3225	165.8625
380.4157	167.3651
380.4589	167.4125
380.8286	167.6805
	167.6955
	168.2999
	168.3193
	171.6074
	171.6580
	171.8168
	172.2967
	172.8595
	173.1216
	173.1280
	173.1458
	173.2375
	173.7806
	173.8174
	173.8851
	173.9016
	173.9044
	173.9336
	174.0551
	174.1419
	174.1832
	174.2225
	174.5292
	174.6954
	174.9728
	175.1123
	175.2171
	175.3236
	175.5528
	175.6109
	176.1028
	176.2785
	176.4961
	176.6580
	176.7312
	176.8020
	176.8781
	176.9136
	177.0508
	177.2486

177.6421
177.8355
178.0047
178.9281
179.0433
179.0909
179.1507
179.5299
179.6019
180.2000
180.4504
193.3457
193.3705
198.8150
198.8786
366.6719
366.6752
366.8664
366.8708
367.0131
367.0726
367.1920
367.2452
367.5607
367.5694
367.6220
367.6329
367.6665
367.8250
367.9987
368.1022
368.3018
368.3540
368.4112
368.6652
370.1463
370.2068
370.9833
371.4378
372.1899
372.8677
376.3945
376.5670
376.7161
376.7183
376.8453
376.8700
376.8740
376.8984

376.9347
377.0372
377.1082
377.2962
377.4341
377.5319
377.5588
377.5938
377.7283
377.7451
377.7522
377.8114
377.8422
377.9968
378.2021
378.3430
378.3662
378.3804
378.4050
378.4305
378.6552
378.6806
379.1553
379.9198
380.0712
381.1898
381.2639
381.4605
382.2481
382.9102
383.4747
384.6191
384.6318
384.8210
385.2894
385.9775
389.3163
389.5615
392.9445
393.1471

CASSCF-SO calculations

CASSCF-SO calculations on complexes **2** and **3** were performed with the program MOLCAS 8.0[20] employing both the XRD-crystal and DFT-optimised structures, with no counterion or solvent molecules. The ANO-RCC basis set library[21] has been used for all the atoms ([ANO-RCC-VTZP] for Dy, [ANO-RCC-VDZP] for coordinating

atoms, and [ANO-RCC-VDZ] for C and H) with scalar relativistic effects accounted using the second-order Douglas–Kroll–Hess transformation. The molecular orbitals (MOs) were optimized in state-averaged CAS(9,7)SCF calculations (9 electrons in the 7 *4f*-orbitals) considering 21, 224 and 490 roots for the sextet, quartet and doublet spin states, respectively. These sets of spin-free states were then used to construct and diagonalize the spin-orbit coupling Hamiltonian in the basis of all sextet, 128 quartet and 130 doublets with the RASSI module. The crystal field decomposition of the ground J = 15/2 multiplet of the ^{6}H term was performed with the SINGLE_ANISO[22] module.

TAB. S6. Electronic structure of **2** and **3** calculated with CASSCF-SO using the crystal geometries. Each row corresponds to a Kramers doublet.

Energy (meV)	Energy (K)	g1	g2	g3	Angle	Wavefunction	<Jz>
				**2**			
0.00	0.00	0.00	0.00	19.88	--	$89\%\vert\pm 15/2\rangle + 11\%\vert\mp 15/2\rangle$	-5.9
51.66	599.53	0.01	0.01	16.99	0.56	$98\%\vert\pm 13/2\rangle$	6.3
77.60	900.54	3.41	4.45	13.30	88.50	$57\%\vert\mp 1/2\rangle + 15\%\vert\pm 1/2\rangle + 14\%\vert\mp 11/2\rangle$	-0.7
82.18	953.66	1.30	4.53	9.33	15.73	$55\%\vert\pm 11/2\rangle + 13\%\vert\pm 3/2\rangle + 11\%\vert\mp 11/2\rangle + 11\%\vert\mp 3/2\rangle + 6\%\vert\pm 1/2\rangle$	-2.5
83.72	971.55	1.58	5.06	9.23	82.86	$49\%\vert\pm 3/2\rangle + 14\%\vert\mp 3/2\rangle + 11\%\vert\mp 1/2\rangle + 9\%\vert\mp 11/2\rangle + 6\%\vert\pm 1/2\rangle$	0.1
89.14	1034.37	0.68	2.83	6.93	14.46	$63\%\vert\pm 5/2\rangle + 20\%\vert\mp 5/2\rangle + 6\%\vert\pm 9/2\rangle$	1.3
94.01	1090.88	0.62	2.72	12.36	38.08	$60\%\vert\pm 9/2\rangle + 7\%\vert\pm 5/2\rangle + 26\%\vert\pm 7/2\rangle$	3.6
95.02	1102.62	0.18	3.43	13.36	45.09	$41\%\vert\pm 7/2\rangle + 24\%\vert\mp 7/2\rangle + 5\%\vert\mp 9/2\rangle + 25\%\vert\pm 9/2\rangle$	-1.6
				**3**			
0.00	0.00	0.00	0.00	19.87	--	$98\%\vert\pm 15/2\rangle$	-7.5
54.21	629.08	0.01	0.01	16.99	1.54	$95\%\vert\pm 13/2\rangle$	6.5
89.53	1038.99	1.09	2.59	13.01	16.25	$80\%\vert\pm 11/2\rangle + 7\%\vert\pm 7/2\rangle + 7\%\vert\pm 3/2\rangle$	4.9

99.10	1149.95	1.86	4.65	12.15	82.20	17%|∓ 1/2⟩ + 13%|± 1/2⟩ + 13%|±9/2⟩ + 10%|±3/2⟩ + 10%|± 5/2⟩ + 11%|∓ 5/2⟩ + 11%|∓ 9/2⟩	0.7
112.42	1304.58	3.13	5.05	9.52	88.64	42%|± 9/2⟩ + 14%|∓ 7/2⟩ + 14%|∓ 3/2⟩ + 8%|∓ 9/2⟩ + 5%|± 3/2⟩	1.1
128.08	1486.30	0.45	0.74	13.74	89.15	36%|± 7/2⟩ + 20%|∓ 9/2⟩ + 15%|∓ 5/2⟩ + 9%|∓ 1/2⟩ + 8%|∓ 7/2⟩	-0.1
145.58	1689.38	0.21	0.30	16.72	89.97	33%|± 5/2⟩ + 22%|∓ 1/2⟩ + 22%|∓ 7/2⟩ + 6%|∓ 5/2⟩	-0.1
155.10	1799.85	0.02	0.07	19.41	88.17	44%|∓ 1/2⟩ + 30%|± 3/2⟩ + 14%|∓ 5/2⟩	0.0

TAB. S7. Electronic structure of **2** and **3** calculated with CASSCF-SO using the PBE optimised geometries. Each row corresponds to a Kramers doublet.

Energy (meV)	Energy (K)	g1	g2	g3	Angle	Wavefunction	<Jz>
				**2**			
0.00	0.00	0.00	0.00	19.89	--	95%|± 15/2⟩ + 5%|∓ 15/2⟩	6.8
51.97	603.08	0.00	0.00	17.02	0.34	94%|± 13/2⟩ + 5%|∓ 13/2⟩	5.8
83.68	971.08	1.27	1.59	13.18	1.65	89%|± 11/2⟩ + 9%|± 1/2⟩	4.8
89.87	1042.87	1.96	4.20	12.73	89.21	69%|± 1/2⟩ + 14%|∓ 1/2⟩ + 8%|± 7/2⟩	0.5
93.23	1081.89	3.61	3.81	5.14	81.67	58%|± 3/2⟩ + 34%|∓ 3/2⟩	-0.3
98.07	1138.10	0.02	1.59	7.35	5.81	67%|± 5/2⟩ + 12%|∓ 5/2⟩ + 15%|± 9/2⟩	-1.9
99.72	1157.24	0.51	0.96	11.08	20.71	75%|± 9/2⟩ + 17%|± 5/2⟩	3.8
102.26	1186.68	0.49	1.23	10.56	30.50	52%|± 7/2⟩ + 42%|∓ 7/2⟩	-0.4
				**3**			
0.00	0.00	0.00	0.00	19.85	--	85%|± 15/2⟩ + 13%|∓ 15/2⟩	5.4
48.81	566.47	0.02	0.02	17.01	2.76	90%|± 13/2⟩ + 8%|∓ 13/2⟩	-5.5
82.56	958.08	0.26	0.49	14.16	14.92	93%|± 11/2⟩	5.3

95.08	1103.34	2.47	3.06	14.77	77.82	$20\%\lvert\pm 1/2\rangle + 18\%\lvert\pm 9/2\rangle + 12\%\lvert\pm 5/2\rangle + 9\%\lvert\mp 1/2\rangle + 9\%\lvert\mp 3/2\rangle + 7\%\lvert\mp 9/2\rangle + 7\%\lvert\pm 3/2\rangle$	0.8
105.30	1221.98	3.26	6.22	8.07	89.21	$50\%\lvert\pm 9/2\rangle + 18\%\lvert\mp 3/2\rangle + 14\%\lvert\mp 7/2\rangle + 7\%\lvert\mp 9/2\rangle + 6\%\lvert\pm 1/2\rangle$	1.2
116.99	1357.57	1.51	2.71	12.95	89.65	$50\%\lvert\pm 7/2\rangle + 29\%\lvert\mp 5/2\rangle + 13\%\lvert\mp 9/2\rangle + 6\%\lvert\mp 1/2\rangle$	0.5
130.14	1510.25	0.40	0.65	16.88	89.36	$22\%\lvert\mp 5/2\rangle +17\%\lvert\pm 3/2\rangle+ + 16\%\lvert\pm 5/2\rangle + 15\%\lvert\pm 7/2\rangle + 15\%\lvert\mp 1/2\rangle$	0.1
136.83	1587.82	0.14	0.31	19.26	88.00	$36\%\lvert\mp 1/2\rangle +21\%\lvert\pm 3/2\rangle+ + 14\%\lvert\pm 1/2\rangle + 11\%\lvert\mp 5/2\rangle + 11\%\lvert\mp 3/2\rangle$	-0.1

Dispersive modes in molecular crystals

In a monoatomic 3-dimensional lattice the phonon spectrum has only acoustic modes with three possible polarizations (one “longitudinal” and two “transverse” modes).

Crystals of single-ion magnets (SIMs) like compounds **1**, **2** and **3** are instead periodic structures where the unit cell contains one or more complex molecules and thus a large number of different atoms.

We can imagine to obtain this structure starting from a reference Debye model, or also from a reference 3d monoatomic crystal as “similar” as possible to the actual crystal: the same type of Bravais Lattice as the SIM-crystal, atomic mass equal to the average mass of the SIM unit cell, spacing such that the average density is the same as in the SIM-crystal, and with the 3 phonon branches fitting the acoustic ones of the SIM-crystal. We can then imagine to smoothly perturb this monoatomic crystal and eventually revert it into the actual one by moving atoms, varying masses and harmonic couplings.

As soon as this process starts, the breaking of periodicity turns a group of unit cells of the monoatomic crystal into the unit cell of the SIM-crystal, and the associated modification of the Brillouin zone (BZ) boundaries in reciprocal space leads to folding of the old acoustic branches into the reduced BZ. The new resulting “optical” modes (i.e., modes whose energy does not tend to zero for q → 0), at zeroth-order are just a relabelling of the former three acoustic modes. But as the reversion proceeds, gaps in the phonon spectrum at the folding points open up and the displacement patterns are modified into those of the SIM-crystal. However, this modification will be minimal for the acoustic branches, and relatively small for low-energy “optical” modes, which are the evolution of old acoustic modes of relatively small q. These low-energy modes represent collective vibrations of the SIM with small bond stretching, and are thus expected to be the less modified by the “perturbation”. For this reason they can be called “pseudoacoustic” modes. Moving to high energies, the folding involves acoustic phonons of large q, and these will be strongly perturbed to eventually nearly match those of the isolated molecule

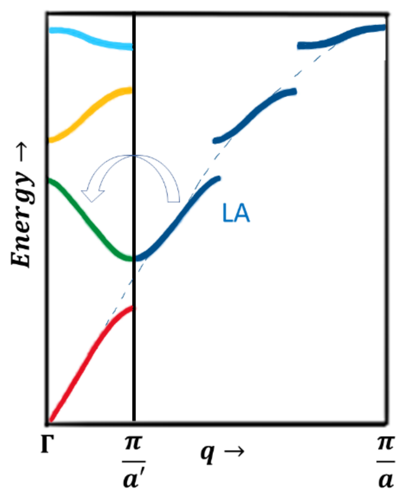

FIG. S12. Example of the folding of a longitudinal acoustic phonon (LA) of a 3d monoatomic lattice with into the reduced Brillouin zone.

Spin-phonon couplings in the Orbach regime

In the Orbach regime the phonon-induced transitions between the 16 crystal field (CF) states of the J = 15/2 multiplet are modelled within a master-equation approach by calculating the rate matrix **W**, which contains transition probabilities between eigenstates of the magnetic terms of the Hamiltonian in eqn 1 induced by the terms H_{Jp} . The rate matrix is calculated as:

$$W_{t\rightarrow s} = \frac{2\pi}{\hbar}\zeta^2\rho(\Delta E_{st})n(\Delta E_{st})\left|\langle s|V_1^{\Gamma}|t\rangle\right|^2,$$

where $\Delta E_{st} = E_s - E_t$, the energy difference between the two eigenstates $|s\rangle$ and $|t\rangle$ of the Hamiltonian H_J, $\rho(\Delta E_{st})$ is the phonon DOS at the frequency corresponding to the gap $\rho(\Delta E_{st})$, $n(x) = (e^{\frac{x}{K_BT}} - 1)^{-1}$ is the Bose-Einstein factor. The potential $V_1^{\Gamma} = \sum_{k,q}\eta_{\Gamma}^{kq}O_k^q$ describes the modulation of the CF induced by the phonon modes, where the coefficients $\eta_{\Gamma}^{kq}$are obtained from ab initio calculations by considering the derivatives of the Steven's operators equivalents coefficients B_k^q , induced by atomic displacements of the ligands along the normal mode Γ computed in the gas-phase approximation. In this work, for each gap ΔE_{st}, we consider all the phonon modes within an energy window of Δ = ±1 meV and we evaluate the average coupling. The values of Δ falls in between the FWHM of the experimental data measured on MERLIN with E_i = 25-27 meV and E_i = 54 meV. This approach guarantees to take into account eventual discrepancies between the calculated energy gaps ΔE_{st} and the resonant phonon modes, both calculated ab initio techniques but with different approaches.

Inelastic Neutron Scattering

The INS experiment on compounds **2** and **3** was performed on the MERLIN spectrometer at the ISIS Neutron and Muon Source[23]. MERLIN is a high-flux time-of-light direct spectrometer operating over a wide range of incident energies.

We measured 2g of polycrystalline sample of both compounds and their non-magnetic Yttrium analogues. We used aluminum sample-holder cans with a thin annular gap to minimize the absorption. Measurements were performed at T = 5 K with three different incident energies in repetition rate multiplication (RRM) mode:

E_i = 90, 130 and 180 meV (maximum incident energy available when using the Gd Fermi chopper). The RRM mode allowed us to measure simultaneously up to 4 separate incident energies each and to collect spectra with optimized experimental resolution for each energy range.

The separation between magnetic and non-magnetic signals was performed by subtracting from the Dy sample data the Y variant ones (see Fig.S13), allowing us to detected for compound **2** a magnetic transition at 62 meV between the ground and the first excited doublets. The magnetic origin of this transition is confirmed by the Q dependence of its intensity (see Fig.S13), which differs from the typical Q^2 behaviour of phononic excitations. This is the only magnetic transition expected for compound **2** in the explored energy range. We expected to observe a similar magnetic excitation for compound **3**, as predicted by CASSCF-SO calculations, but was not detectable being covered by phonon modes at around 60 meV.

The full neutron-weighted density of states (nw-DOS) over the whole explored energy range was reconstructed by merging the results of several INS datasets with different experimental configurations and incident energies. Thus, we normalized each dataset by the incident neutron flux at the corresponding energy measured by the beam monitor before the sample. We treated the data with the Mantid software,[24] which allowed us to extract the nw-DOS from the measured INS data on powders in the one-phonon incoherent approximation to be compared with DFT calculations.

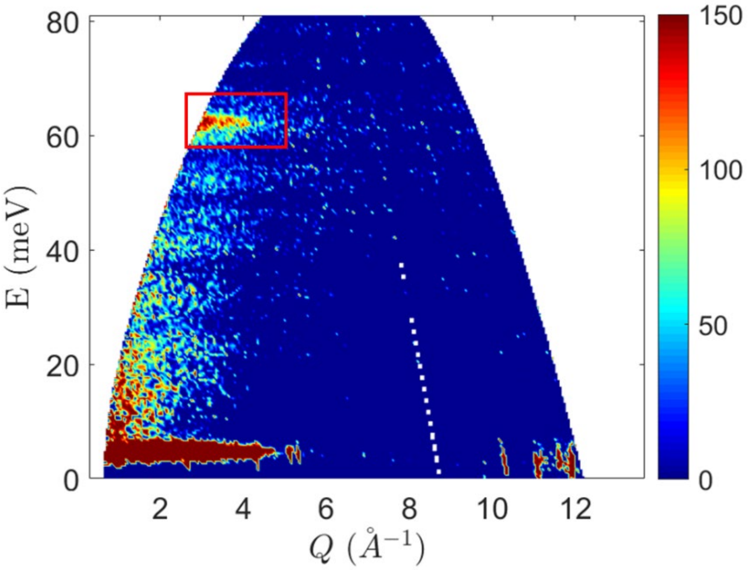

FIG. S13. Powder INS spectrum of **2** in the (*Q, E*) space, after subtraction of the signal of its diamagnetic Y analogue, measured on the MERLIN spectrometer with incident energy 90 meV. The monotonic decrease of the intensity of the peak at about 62 meV as a function of *Q* (highlighted by the red box) demonstrates its magnetic origin.

Incoherent approximation

The inelastic scattering function S(Q,E) measured on powder samples is generally interpreted within the so-called incoherent approximation. Within this approach the S(Q,E) is proportional to the neutron-weighted DOS:

$$S_{inc}(Q,E) \propto \frac{Q^2}{E}\left[\sum_i \frac{\sigma_i^{scatt}}{2m_i}\rho_i(E)\right],$$

where $\rho_i(E)$ is the partial DOS for each element *i*, weighted by its scattering cross-section σ_i^{scatt} and its atomic mass m_i. Q and E are the momentum and energy transfer of the neutron. To reproduce the measured INS data, we performed periodic DFT simulations to calculate the partial DOS for each element and reconstruct the neutron-weighted DOS.

Axiality of the crystal-field eigenstates

The *axiality* of the crystal-field eigenstate $|\psi_i\rangle$ is defined as $|\langle m_J|\psi_i\rangle|^2$ and measures the component of the eigenstate on the m_J state with the largest component. The *rhombicity* therefore defined as $1 - |\langle m_J|\psi_i\rangle|^2$. When averaged, this quantities are averaged over all the $|m_J|$ doublets of the J = 15/2 multiplet. Table S8 report these values for the J = 15/2 doublets of compounds 1, 2 and 3.

TAB. S8. Rhombicity of all the doublets of the J = 15/2 multiplet for compounds 1, 2 and 3. The last two lines report the average rhombicity and axiality.

**Doublet**	**2**	**3**	**1**
Ground state	0.0004	0.0128	0.0064
1st excited	0.0029	0.0172	0.0046
2nd excited	0.3165	0.1036	0.0072
3rd excited	0.3799	0.75	0.0097
4th excited	0.0813	0.4263	0.0163
5th excited	0.0947	0.586	0.0287
6th excited	0.1226	0.7506	0.0965
7th excited	0.0746	0.6519	0.1049
Average rhombicity	0.1341	0.4123	0.034288
Average axiality	0.8658	0.5877	0.965713

Comparison of effective coefficients with literature

TAB. S9. Comparison between effective parameters of Raman and Orbach processes extracted from our calculations (in the temperature range specified in the main text) and fitted from experiments (Refs. 2,25, in brackets).

	**2**	**3**	**1**
n	3.6 ± 0.2	3.0 ± 0.1	2.3
	(4.6)	(2.86)	(2.2)
C	$(5 \pm 3) \times 10^{-5}$	$(3 \pm 1) \times 10^{-3}$	4×10^{-7}
	(2×10^{-6})	(4.5×10^{-3})	(1.6×10^{-6})
U_{eff}	1093	1127	1786
	(950)	(1210)	(1760)
τ_0	$(1.5 \pm 0.5) \times 10^{-13}$	$(3 \pm 2) \times 10^{-12}$	1.8×10^{-11}
	(3×10^{-12})	(7.9×10^{-13})	(1.99×10^{-11})

References

(1) Ding, Y.; Han, T.; Zhai, Y.; Reta, D.; Chilton, N. F.; Winpenny, R. E. P.; Zheng, Y., *Chem. Eur. J.* **2020**, *26* (26), 5893–5902.

(2) Y.-S. Ding et al., Nat. Commun. **9**, 3134 (2018); Parmar, V. S.; Ortu, F.; Ma, X.; Chilton, N. F.; Clérac, R.; Mills, D. P.; Winpenny, R. E. P., *Chem.Eur. J.* **2020**, *26* (35), 7774–7778.

(3) Michiue, K.; Jordan, R. F., *J. Mol. Catal. A Chem.* **2008**, *282* (1–2), 107–116.

(4) Huang, W.; Le Roy, J. J.; Khan, S. I.; Ungur, L.; Murugesu, M.; Diaconescu, P. L., *Inorg. Chem.* **2015**, *54* (5), 2374–2382.

(5) Agilent. CrysAlisPro Data Collection and Processing Software for Agilent X-Ray Diffractometers. *Technol. UK Ltd, Yarnton, Oxford, UK* **2014**, *44* (0), 1–53.

(6) Sheldrick, G. M., *Acta Crystallogr. Sect. A Found. Crystallogr.* **2008**, *64* (1), 112–122.

(7) Sheldrick, G. M., *Acta Crystallogr. Sect. C Struct. Chem.* **2015**, *71* (1), 3–8.

(8) Dolomanov, O. V.; Bourhis, L. J.; Gildea, R. J.; Howard, J. A. K.; Puschmann, H., *J. Appl. Crystallogr.* **2009**, *42* (2), 339–341.

(9) Farrugia, L. J., *J. Appl. Crystallogr.* **2012**, *45* (4), 849–854.

(10) Persistence of Vision Raytracer (Version 3.6). POV-Ray. Persistence of Vision Pty. Ltd. (2004): Williamstown, Victoria, Australia. 2004.

(11) Giannozzi P, Baseggio O., Bonfà P., Brunato D. , Car R., Carnimeo I., Cavazzoni C., de Gironcoli S., Delugas P., Ruffino F.F., Ferretti A., Marzari N., Timrov I., Urru A. and Baroni S., *J. Chem. Phys.*, 2020, **152**, 154105

(12) Garrity K. F., Bennett J. W., Rabe K. M. and Vanderbilt D., *Comput. Mater. Sci.*, 2014, **81**, 446.

(13) (a) Perdew J. P., Burke K. and Ernzerhof M., *Phys. Rev. Lett.*, 1996, **77**, 3865. (b) Perdew J. P., Burke K. and Ernzerhof M., Phys. Rev. Lett. 1997, **78**, 1396–1396.

(14) Togo A:, Oba F. and Tanaka I., *Phys. Rev. B*, 2008, **78**, 134106.

(15) Skelton J. M., Parker S. C., Togo A., Tanaka I. and Walsh A., *Phys. Rev. B*, 2014, **89**, 205203.

(16) Frisch M. J., et al. Gaussian 09, Revision D.01, Gaussian, Inc., Wallingford CT, 2016.

(17) Dunning T. H., *J. Chem. Phys.* 1989, **90**, 1007–1024.

(18) Martin J. M. L. and Sundermann A., *J. Chem. Phys*. 2001, **114**, 3408–3420.

(19) (a) Grimme S., *J. Comput. Chem.* 2004, **25** (12), 1463–1473. (b) Grimme S., *J. Comput. Chem.* 2006, **27** (15), 1787–1799. (c) Grimme S., Wiley *Interdiscip. Rev. Comput. Mol. Sci.* 2011, **1** (2), 211–228.

(20) Aquilante, F. et al, *J. Comput. Chem.* 2016, 37, 506-541.

(21) (a) Roos, B. O.; Lindh, R.; Malmqvist, P.-Å.; Veryazov, V.; Widmark, P.-O. *J. Phys. Chem. A* 2005, **109**, 6575–6579.
(b) Roos, B. O.; Lindh, R.; Malmqvist, P.-Å.; Veryazov, V.; Widmark, P.-O. *J. Phys. Chem. A* 2004, **108**, 2851–2858.

(22) Ungur L., Chibotaru L. F. *Chem. Eur. J.* 2017, **23**, 3708–3718.

(23) Bewley R., Eccleston R., McEwen K., Hayden S., Dove M., Bennington S., Treadgold J.and Coleman R., *Physica B*, 2006, **385-386**, 1029–1031.

(24) Arnold O., Bilheux J.C., Borreguero J.M., Buts A., Campbell S.I., Chapon L., Doucet M., Draper N., Ferraz Leal R., Gigg M.A., Lynch V.E., Markvardsen A., Mikkelson D.J., Mikkelson R.L., Miller R., Palmen K., Parker P., Passos G., Perring T.G., Peterson P.F., Ren S., Reuter M.A., Savici A.T., Taylor J.W., Tolchenov R., Zhou W., Zikovsky J., *Nuclear Instruments and Methods in Physics Research* A, 2014, **764**, 156166.

(25) C. A. P. Goodwin, F. Ortu, D. Reta, N. F. Chilton & D. P. Mills, Nature **548**, 439 (2017).
